# Two-Year Efficacy and Safety of Mirikizumab Following 104 Weeks of Continuous Treatment for Ulcerative Colitis: Results From the LUCENT-3 Open-Label Extension Study

**DOI:** 10.1093/ibd/izae024

**Published:** 2024-03-09

**Authors:** Bruce E Sands, Geert D’Haens, David B Clemow, Peter M Irving, Jordan T Johns, Theresa Hunter Gibble, Maria T Abreu, Scott Lee, Tadakazu Hisamatsu, Taku Kobayashi, Marla C Dubinsky, Severine Vermeire, Corey A Siegel, Laurent Peyrin-Biroulet, Richard E Moses, Joe Milata, Vipin Arora, Remo Panaccione, Axel Dignass

**Affiliations:** Dr. Henry D. Janowitz Division of Gastroenterology, Icahn School of Medicine at Mount Sinai, New York, NY, USA; Amsterdam University Medical Centers, Amsterdam, the Netherlands; Eli Lilly and Company, Indianapolis, IN, USA; Guy’s and St. Thomas’ NHS Foundation Trust, King’s College London, London, United Kingdom; Eli Lilly and Company, Indianapolis, IN, USA; Eli Lilly and Company, Indianapolis, IN, USA; UHealth Crohn’s and Colitis Center, University of Miami Miller School of Medicine, Miami, FL, USA; Digestive Health Center, University of Washington Medical Center, Seattle, WA, USA; Department of Gastroenterology and Hepatology, Kyorin University School of Medicine, Tokyo, Japan; Center for Advanced IBD Research and Treatment, Kitasato Institute Hospital, Kitasato University, Tokyo, Japan; Icahn School of Medicine at Mount Sinai, New York, NY, USA; Department of Gastroenterology and Hepatology, UZ Leuven, Leuven, Belgium; Dartmouth Hitchcock Medical Center, Lebanon, NH, USA; Department of Gastroenterology, INFINY Institute, FHU-CURE, French Institute of Health and Medical Research Nutrition–Genetics and Exposure to Environmental Risks Research Unit, Nancy University Hospital, Nancy, France; Paris IBD Center, Groupe Hospitalier Privé Ambroise Paré–Hartmann, Neuilly-sur-Seine, France; Division of Gastroenterology and Hepatology, McGill University Health Centre, Montreal, QC, Canada; Eli Lilly and Company, Indianapolis, IN, USA; Eli Lilly and Company, Indianapolis, IN, USA; Eli Lilly and Company, Indianapolis, IN, USA; Inflammatory Bowel Disease Group, Cumming School of Medicine, University of Calgary, Calgary, AB, Canada; Department of Medicine I, Agaplesion Markus Krankenhaus, Frankfurt, Germany

**Keywords:** mirikizumab, ulcerative colitis, IL-23 p19 antibody, long-term extension, week 104 results

## Abstract

**Background:**

Mirikizumab, a p19-directed interleukin-23 monoclonal antibody, is efficacious in inducing clinical remission at week 12 (W12) and maintaining clinical remission at W52 in patients with moderately to severely active ulcerative colitis. Results are presented from the open-label extension study through W104.

**Methods:**

Clinical, symptomatic, quality-of-life, and adverse event outcomes are reported for mirikizumab induction responders and extended induction responders, including biologic-failed patients, who entered LUCENT-3, with data shown for W52 maintenance responders or remitters. Discontinuations or missing data were handled by nonresponder imputation (NRI), modified NRI (mNRI), and observed case (OC).

**Results:**

Among W52 mirikizumab responders, clinical response at W104 was 74.5%, 87.2%, and 96.7% and clinical remission was 54.0%, 62.8%, and 70.1% for NRI, mNRI, and OC, respectively. Among W52 mirikizumab remitters, clinical response at W104 was 76.6%, 89.0%, and 98.3% and clinical remission was 65.6%, 76.1%, and 84.2%. Using mNRI, remission rates at W104 for W52 clinical remitters were 74.7% corticosteroid-free, 79.5% endoscopic, 63.9% histologic-endoscopic mucosal remission, 85.9% symptomatic, 59.8% bowel urgency, 80.5% Inflammatory Bowel Disease Questionnaire (using NRI), 71.2% histologic-endoscopic mucosal improvement, and 77.5% bowel urgency improvement. Previous biologic-failed vs not-biologic-failed patient data were generally similar. Extended induction mNRI clinical response was 81.9%. Serious adverse events were reported in 5.2% of patients; 2.8% discontinued treatment due to adverse events.

**Conclusions:**

Endoscopic, histologic, symptomatic, and quality-of-life outcomes support the long-term benefit of mirikizumab treatment up to 104 weeks in patients with ulcerative colitis, including biologic-failed patients, with no new safety concerns.

**Key Messages**

*What is already known?* Mirikizumab, a p19-directed interleukin-3 monoclonal antibody, is effective at 12 weeks of induction and 52 weeks of maintenance treatment in patients with moderately to severely active ulcerative colitis.

*What is new here?* Long-term treatment with mirikizumab up to 104 weeks is associated with a sustained and durable effect on clinical response/remission, endoscopic, histologic, symptomatic, and quality-of life outcomes in patients who had previously failed biologic therapy and those who had not.

*How can this study help patient care?* This study provides long-term (2-year) treatment data for mirikizumab, informing benefit-risk decisions when prescribing this new biologic to patients with moderately to severely active ulcerative colitis.

## Introduction

Ulcerative colitis (UC) is a recurring and remitting inflammatory disease of the rectum and colon that negatively impacts quality of life.^1^ Treatment goals include symptom control, induction of response, and maintenance of remission with the aim of achieving endoscopic remission and modifying the course of the disease so that disease-related disability is minimized and patients can achieve an improved quality of life.^2^ Although several categories of medications are available, ranging from corticosteroids, mesalamines, immunosuppressors, sphingosine-1 phosphate modulators, Janus kinase inhibitors, and biologics, patients with moderately to severely active UC still have an unmet need for treatment options.^3^

Despite considerable advances in expanding treatment options, in practice, gaining a satisfactory outcome for all patients with UC remains elusive.^4-6^ Many patients continue to be burdened by physical and psychological symptoms that impact quality of life, and a substantial number of patients still require restorative proctocolectomy.^7^ There is a need to achieve not only control of clinical symptoms, but also mucosal healing such as histologic-endoscopic mucosal remission (HEMR) to attain the best possible long-term outcomes. Major unmet needs include sustained corticosteroid-free remission, HEMR, bowel urgency remission, and normalization of physical and psychological quality of life.

Interleukin (IL)-23 plays an important role in the pathogenesis of UC through promotion of a T helper 17 cell–related immune response.^8,9^ Mirikizumab is a specific blocker of IL-23 through the p19 subunit of the IL-23 molecule and is the first approved antibody therapy in its class for UC. Mirikizumab is a humanized monoclonal antibody that has demonstrated sustainable efficacy in patients with moderately to severely active UC, in whom conventional or advanced therapies have failed (LUCENT-1 [NCT03518086], LUCENT-2 [NCT03524092]).^10-12^

Here, we present findings from LUCENT-3, which examined the long-term efficacy and safety of mirikizumab in patients who completed 104 weeks of continuous treatment with mirikizumab.

## Methods

### Study Oversight

All patients involved in this study provided informed consent. The protocol, amendments, and consent documentation were approved by local ethical review boards. The study was registered at the European Network of Centers for Pharmacoepidemiology and Pharmacovigilance and was conducted in accordance with the Declaration of Helsinki and International Council for Harmonization of Technical Requirements for Pharmaceuticals for Human Use guidelines, including Good Clinical Practices and Good Pharmacoepidemiology Practices.^13^ An independent data monitoring committee monitored LUCENT-1, LUCENT-2, and LUCENT-3. The trials were registered at Clinical Trials.gov (NCT03518086, NCT03524092, and NCT03519945, respectively).

### Study Design

**Figure 1.**
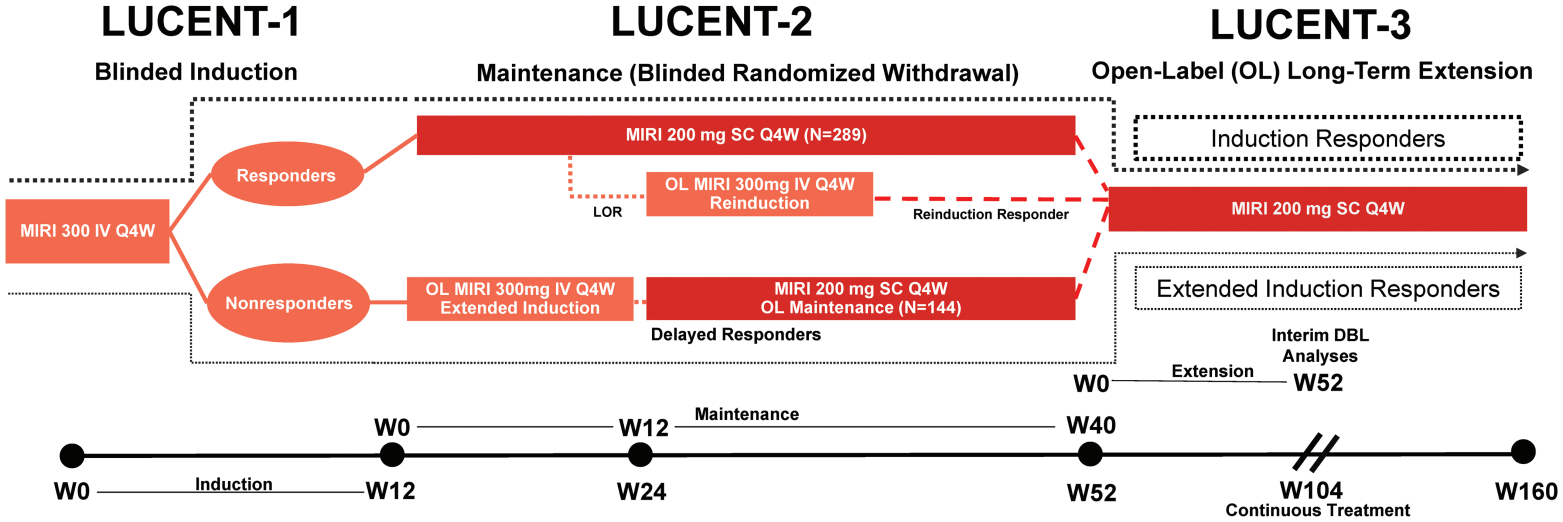
LUCENT clinical trial program with LUCENT-3 patient flow pathways for analyzed populations. Response is defined as achieving ≥2-point and ≥30% decrease in the modified Mayo score (MMS) from induction baseline with rectal bleeding (RB) = 0 or 1, or ≥1-point decrease from baseline. DBL, data base lock; IV, intravenous; LOR, loss of response; MIRI, mirikizumab; OL, open-label; Q4W, every 4 weeks; SC, subcutaneous; W, week.

The study design and treatment protocols of the 12-week LUCENT-1 induction study and the 40-week LUCENT-2 maintenance study have been previously described.^10^ LUCENT-3 (NCT03519945) is an ongoing single-arm, open-label, phase 3, multicenter, long-term extension study evaluating the efficacy and safety of mirikizumab in patients with moderately to severely active UC who participated in the LUCENT-1 induction study and LUCENT-2 maintenance study (Figure 1). In LUCENT-1, patients received mirikizumab 300 mg intravenously (IV) at weeks 0, 4, and 8. In LUCENT-2, mirikizumab induction responders received 200 mg every 4 weeks subcutaneously from week 12 (week 0 of maintenance) through week 52. From week 12, induction nonresponders received extended induction with mirikizumab 300 mg IV at weeks 12, 16, and 20; extended induction responders received an additional 200 mg mirikizumab every 4 weeks subcutaneously from week 24 (week 12 of maintenance). Mirikizumab induction responders who experienced a loss of treatment response received reinduction, with 3 doses of mirikizumab 300 mg IV every 4 weeks, after which patients who demonstrated benefit from therapy based on investigator opinion were returned to 200 mg mirikizumab every 4 weeks subcutaneously by entering LUCENT-3 (reinduction responders). In LUCENT-3, all patients received 200 mg mirikizumab every 4 weeks subcutaneously. Other patient cohorts included in the LUCENT studies not currently discussed have been previously described.^10-12^

The key inclusion criterion for LUCENT-3 was patients from the phase 3 maintenance study LUCENT-2 who completed the week 52 (week 40 LUCENT-2) visit on blinded subcutaneous therapy and, per investigator opinion, would benefit from continuing treatment with mirikizumab in LUCENT-3. Weeks are shown as cumulative; week 12 of LUCENT-2 is defined as week 24 overall, such that LUCENT-2 week 40 is equivalent to 52 weeks of continuous treatment, and LUCENT-3 week 52 is 104 weeks of continuous treatment.

Loss of response rescue could not be administered until the patient received at least 12 weeks of blinded maintenance therapy; thus, these patients received 12 weeks of treatment in LUCENT-1 (induction), at least 12 weeks in LUCENT-2 (maintenance), and 12 weeks of reinduction treatment in LUCENT-2, constituting a minimum of 36 weeks of mirikizumab in LUCENT-1 and LUCENT-2 prior to entering LUCENT-3. Because these patients rolled over into LUCENT-3 directly after reinduction treatment, they may not have had 104 weeks of continuous treatment when assessed at week 52 of LUCENT-3.

### Patient Groups

For the LUCENT clinical program modified intention-to-treat population (n = 1162), 868 patients were randomized to receive mirikizumab induction treatment in LUCENT-1. Of these patients, 816 entered the LUCENT-2 maintenance study—544 entered as mirikizumab induction responders and 272 as mirikizumab induction nonresponders who at first received extended induction treatment.

Among the mirikizumab induction responders, 365 were rerandomized to continue mirikizumab treatment maintenance therapy and 324 completed 52 weeks of treatment.

Among the mirikizumab induction nonresponders, 134 were delayed responders and completed 52 weeks of treatment.

Of the LUCENT-2 week 52 completers that entered into LUCENT-3 and were eligible for the current LUCENT-3 interim analyses, 266 mirikizumab induction responders and 102 extended induction responders are included.

Several populations are relevant for the current analyses:

- *Induction responders*: LUCENT-1 induction week 12 mirikizumab responders who stayed on blinded mirikizumab in LUCENT-2 maintenance and continued to LUCENT-3; main analysis cohort (blinded maintenance completers).
- *Extended induction responders*: Patients from LUCENT-2 who received and responded to extended induction mirikizumab treatment and completed the LUCENT-2 week 40 (week 52 continuous treatment) visit on open-label mirikizumab treatment.
- *Maintenance remitters*: Induction responders who were then LUCENT-2 week 40 (week 52 continuous mirikizumab treatment) clinical remitters.
- *Maintenance responders*: Induction responders who were then LUCENT-2 week 40 (week 52 continuous mirikizumab treatment) clinical responders.
- *Modified intention-to-treat population:* All patients who received any study treatment during this study; excluding patients impacted by an electronic clinical outcome assessment transcription error in Poland and Turkey,^10^ regardless of whether the patient did not receive the correct treatment, or otherwise did not follow the protocol.
- *Reinduction responders*: Induction responders from LUCENT-1 who had a loss of response during LUCENT-2, who received reinduction treatment during LUCENT-2 and who were benefiting from mirikizumab treatment in the opinion of the investigator, and who then moved to LUCENT-3; this does not include patients randomized to placebo in LUCENT-2.
- *Safety*: All patients who received any amount of study treatment, regardless of whether the patient did not receive the correct treatment or otherwise did not follow the protocol.

Induction baseline biologic-failed and not-biologic-failed subgroups were also analyzed:

- *Biologic failed*: As of LUCENT-1 induction baseline; prior inadequate response, loss of response, or intolerance to biologic therapy or Janus kinase inhibitors (tofacitinib).
- *Not biologic failed:* As of LUCENT-1 induction baseline; patients not meeting the biologic-failed definition who had, however, failed a conventional therapy such as immunomodulators or corticosteroids.

Not all patients met the responder or remitter definition at their last visit measurement in LUCENT-2. Thus, the maintenance responder and maintenance remitter subgroups are considered subsets of the induction responder population when moved into LUCENT-3. Only 27 of 266 induction responders did not meet the response criteria at LUCENT-2 week 40 (week 52 of continuous treatment).

No patients on placebo, whether directly from LUCENT-2 or the induction responder population randomly assigned to placebo in LUCENT-2, were included in the analysis.

### Outcome Measures

The primary endpoints and major secondary endpoints for LUCENT-1 and LUCENT-2 have been previously reported.^10^ Endpoints associated with the current analyses are noted here:

- *Abdominal pain ≥30% improvement*: ≥30% change from baseline in Abdominal Pain Numeric Rating Scale score with Abdominal Pain Numeric Rating Scale score ≥3 at baseline.
- *Abdominal pain severity*: Change in Abdominal Pain Numeric Rating Scale score from induction baseline.
- *Alternate clinical remission*: Stool frequency (SF) = 0 or SF = 1; rectal bleeding (RB) = 0; and endoscopic subscore (ES) = 0 or 1 (excluding friability).
- *Bowel urgency clinically meaningful improvement:* Decrease from baseline in Urgency Numeric Rating Scale score ≥3 in patients with Urgency Numeric Rating Scale ≥3 at induction baseline.^14^
- *Bowel urgency remission*: Urgency Numeric Rating Scale = 0 or 1.
- *Bowel urgency severity*: Change in Urgency Numeric Rating Scale score from induction baseline.
- *Clinical remission*: SF = 0 or SF = 1 with ≥1-point decrease in modified Mayo score from baseline; RB = 0; and ES = 0 or 1 (excluding friability).
- *Clinical response*: ≥2-point and ≥30% decrease in modified Mayo score from baseline; RB = 0 or 1, or RB ≥1-point decrease from baseline.
- *Corticosteroid-free remission*: Clinical remission with no corticosteroid use for ≥12 weeks.
- *Endoscopic remission*: ES = 0 or 1 (excluding friability).
- *Histologic-endoscopic mucosal improvement (HEMI):* Geboes ≤3.1 + ES = 0 or 1 (excluding friability); histologic improvement, defined using Geboes scoring system with neutrophil infiltration in <5% of crypts, no crypt destruction, and no erosions, ulcerations, or granulation tissue.
- *HEMR:* Geboes ≤2B.0 + ES = 0 or 1 (excluding friability); histologic remission with resolution of neutrophils, defined using Geboes scoring of ≤2B.0; Geboes subscores of 0 for grades 2b (lamina propria neutrophils), 3 (neutrophils in epithelium), 4 (crypt destruction), 5 (erosion or ulceration).
- *Inflammatory Bowel Disease Questionnaire (IBDQ) remission*: IBDQ total score ≥170.^1^
- *IBDQ response*: ≥16-point improvement from baseline.^1^
- *IBDQ severity*: Change in IBDQ total score and domain scores (bowel symptoms, emotional function, social function, systematic symptoms) from induction baseline.
- *RB severity*: Change in RB modified Mayo score subscore from induction baseline.
- *SF severity*: Change in SF modified Mayo score subscore from induction baseline.
- *Symptomatic remission*: SF = 0 or SF = 1 with ≥1-point decrease in modified Mayo score from baseline; RB = 0.

The baseline for the current analyses was induction baseline from LUCENT-1.

### Statistical Analyses

All endpoints are summarized using descriptive statistics. Categorical efficacy endpoints are summarized using proportions and confidence intervals (CIs), in which CIs are calculated using the Wilson score method,^15,16^ unless otherwise specified. Continuous efficacy endpoints are summarized using mean change from the LUCENT-1 study induction baseline and the standard deviation. Efficacy analyses were performed on the modified intention-to-treat population at the indicated time point. Safety summaries are provided for the safety population and include events or results during weeks 52 to 104 (weeks 0-52, LUCENT-3) of treatment.

### Missing Data Handling

Approximately 25% of patients have missing data at week 104 of LUCENT-3 due to early discontinuation or being sporadically missing (missing at random). For full details, see Missing Data Handling and Importance of Data Interpretation Based Upon Analytical Method (Supplementary Content 1).

For symptom efficacy analyses, missing data were primarily from patient diaries. Five patients had missing endoscopies and 23 patients discontinued early. Sporadic missingness is defined as missing data needed to assess the endpoint due to reasons other than treatment discontinuation, such as not having sufficient days of diary data, or missing an endoscopy. For all patients with sporadically missing observations prior to discontinuation, the last nonmissing observation before the sporadically missing observation was carried forward to the corresponding visit. The safety populations for induction responders and extended induction responders are summarized separately, and include individuals impacted by the electronic clinical outcome assessment transcription error in Poland and Turkey.^10^

Nonresponder imputation was prespecified as the primary approach to handle missing data for all categorical or binary endpoints, and patients who discontinued treatment or were missing endpoint assessments were treated as nonresponders. Nonresponder imputation is a conservative analytical approach and can be biased to show low remission/response rates. For continuous efficacy variables over time, a mixed-effects model for repeated measures was used to estimate the mean change from baseline. Visit was the only additional variable added to the model. For continuous measures at a single time point, modified baseline observation carried forward was applied in the case of missing data. For patients discontinuing mirikizumab due to an adverse event, the baseline observation for the endpoint was carried forward to the corresponding visit for all missing observations after the patient discontinued study treatment. For patients discontinuing mirikizumab for any other reason, the last nonmissing postbaseline observation before discontinuation was carried forward to the corresponding visit for all missing observations. Patients without at least 1 postbaseline observation were not included for evaluation.

Observed case analyses were performed as secondary analyses for categorical data, in which patients with missing data were not included and missing data were not imputed. Observed case analyses can be biased to show high remission/response rates. As such, modified nonresponder imputation was applied; modified nonresponder imputation includes multiple imputation^17^ and is a balance between nonresponder imputation and observed case analyses because it counts treatment discontinuation as nonresponse but addresses sporadic missing data. For modified nonresponder imputation, for patients discontinuing treatment for any reason, nonresponder imputation was used to impute the missing data. For patients having sporadic missing data, multiple imputation was used. Multiple imputation uses logistic regression to make multiple predictions of the missing values and obtains multiple estimations for each of these predictions, providing an imputation value. The percentage of response and the CIs are calculated using Rubin’s rules^17^ to combine multiple imputation datasets. Standard errors for each imputed dataset are calculated using the asymptotic method, without continuity correction. As multiple imputation uses multiple estimates, there is no end result of number of patients in the analyses, but rather there is the estimated proportion responding using the modeling.

## Results

### Baseline Demographics and Disease Characteristics

Baseline demographics for the induction responder population and the extended induction responder population were generally similar with minor exceptions for body mass index, male sex, and Asian descent (Supplementary Table 1).

Disease duration was shorter and there was a lower percentage of pancolitis, severe modified Mayo score, severe endoscopic Mayo subscore, and baseline corticosteroid use in the induction responder population compared with extended induction responder population; lower inflammatory markers (C-reactive protein, fecal calprotectin), prior biologic or tofacitinib failure, and lower overall percentage of failed biologics or tofacitinib were also observed (Supplementary Table 1).

### Efficacy

#### Clinical endpoint outcomes

**Figure 2.**
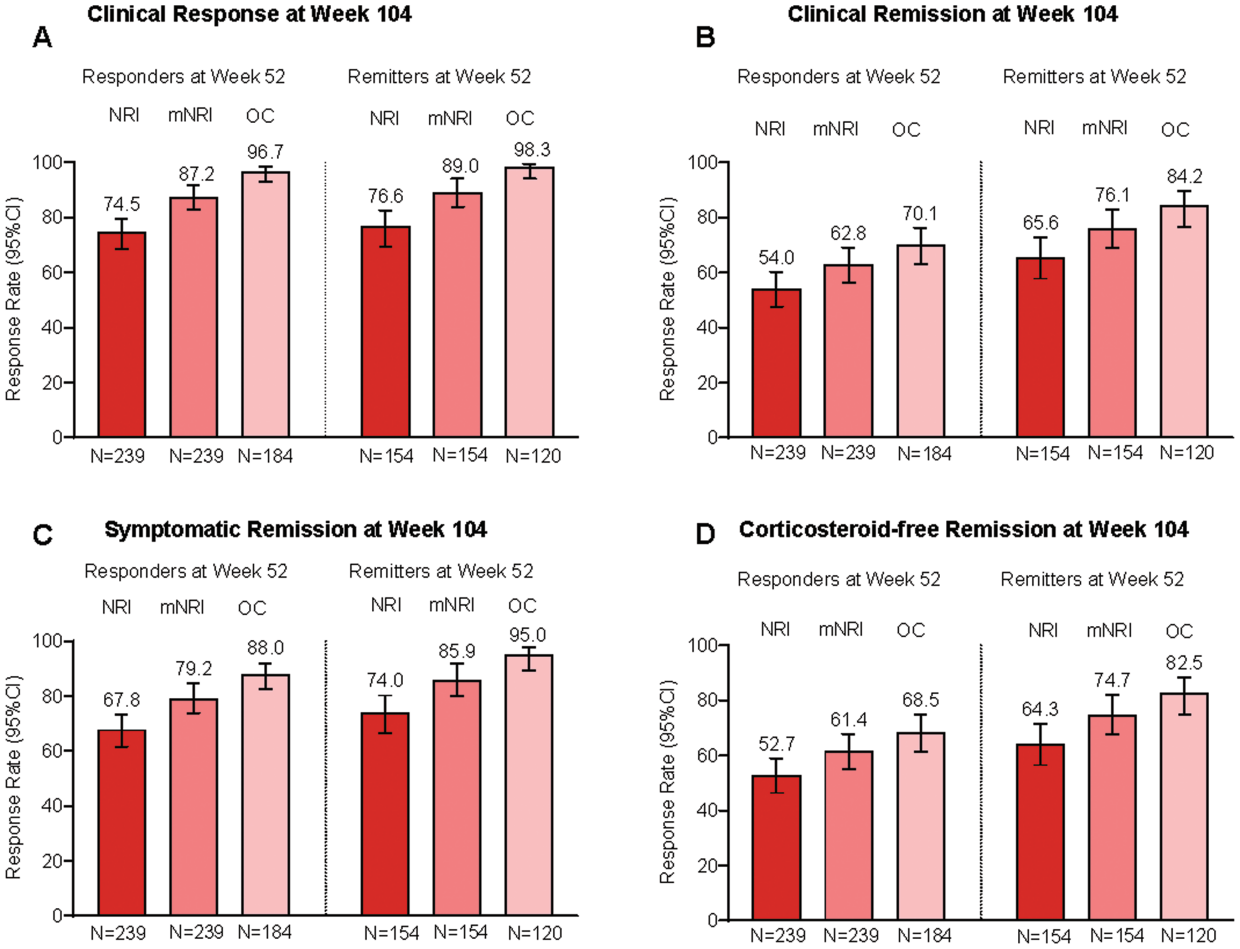
LUCENT-3 rates at 104 weeks of continuous treatment in LUCENT-2 week 52 responders and remitters for (A) clinical response, (B) clinical remission, (C) symptomatic remission, and (D) corticosteroid-free remission, nonresponder imputation (NRI), modified NRI (mNRI), observed case. The modified intention-to-treat population was used with the NRI, mNRI, and observed case methods for missing data. Responders: ≥30% and 2-point decrease from baseline in the composite clinical endpoint of the sum of endoscopic subscore (ES) and stool frequency (SF), and rectal bleeding (RB) subscores, and RB = 0 or 1, or ≥1-point decrease from baseline. Remitters: modified Mayo score SF = 0 or SF = 1 with ≥1-point decrease from baseline; RB = 0; ES = 0 or 1. Symptomatic remission: SF = 0 or SF = 1 with ≥1-point decrease in modified Mayo score from baseline; RB = 0. Corticosteroid-free remission refers to clinical remission with no corticosteroid use for ≥12 weeks. For the mNRI method, for week 52 responders for endpoints: treatment discontinuation, 23 (9.6%); sporadic missing 32 (13.4%). For the mNRI method, for week 52 remitters: treatment discontinuation, 14 (9.1%); sporadic missing 20 (13.0%). CI, confidence interval.

**Figure 3.**
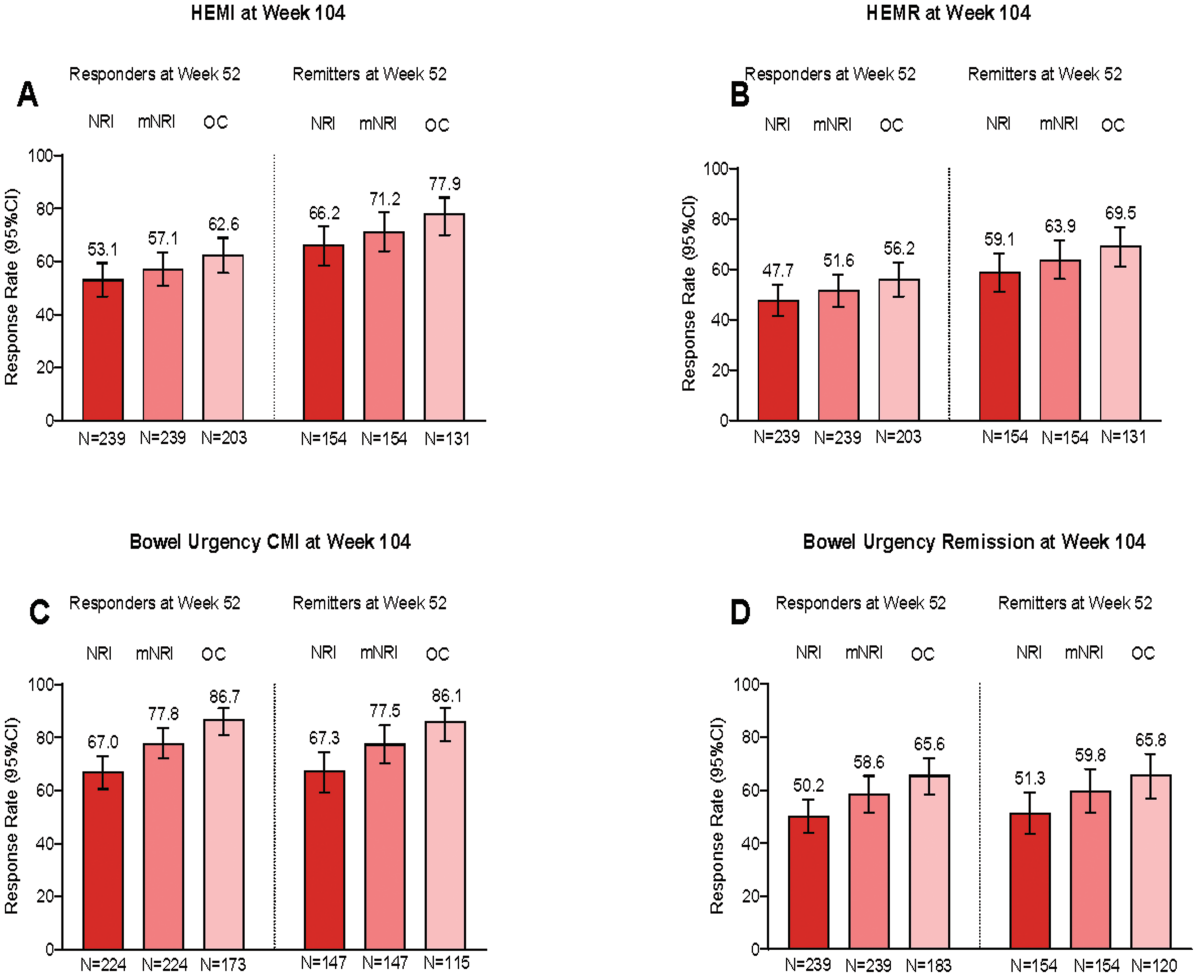
LUCENT-3 rates at 104 weeks of continuous treatment in LUCENT-2 week 52 responders and remitters for (A) histologic-endoscopic mucosal improvement (HEMI) (Geboes ≤3.1 + endoscopic subscore [ES] = 0 or 1 [excluding friability]), (B) histologic-endoscopic mucosal remission (HEMR) (Geboes ≤2B.0 + ES = 0 or 1 [excluding friability]), (C) bowel urgency clinically meaningful improvement (CMI) (change from baseline in Urgency Numeric Rating Scale [NRS] ≥3 in patients with Urgency NRS ≥3 at induction baseline), and (D) bowel urgency remission, nonresponder imputation (NRI), modified NRI (mNRI), observed case. The modified intention-to-treat population was used with the NRI, mNRI, and observed case methods for missing data. Responders: ≥30% and 2-point decrease from baseline in the composite clinical endpoint of the sum of ES and stool frequency (SF) and rectal bleeding (RB) subscores, and RB = 0 or 1, or ≥1-point decrease from baseline. Remitters: modified Mayo score SF = 0 or SF = 1 with 2:1-point decrease from baseline; RB = 0; ES = 0 or 1. Bowel urgency remission: Urgency NRS = 0 or 1. For the mNRI method, HEMI/HEMR- week 52 responders: treatment discontinuation, 23 (9.6%); sporadic missing, 13 (5.4%). For the mNRI method, HEMI/HEMR week 52 remitters: treatment discontinuation, 14 (9.1%); sporadic missing, 9 (5.8%). For the mNRI method, bowel urgency CMI week 52 responders: treatment discontinuation, 22 (9.8%); sporadic missing, 29 (12.9%). For the mNRI method, bowel urgency CMI week 52 remitters: treatment discontinuation, 14 (9.5%); sporadic missing, 18 (12.2%). For the mNRI method, bowel urgency remission week 52 responders: treatment discontinuation, 23 (9.6%); sporadic missing, 33 (13.8%). For the mNRI method, bowel urgency remission week 52 remitters: treatment discontinuation, 14 (9.1%); sporadic missing, 20 (13.0%). CI, confidence interval.

**Figure 4.**
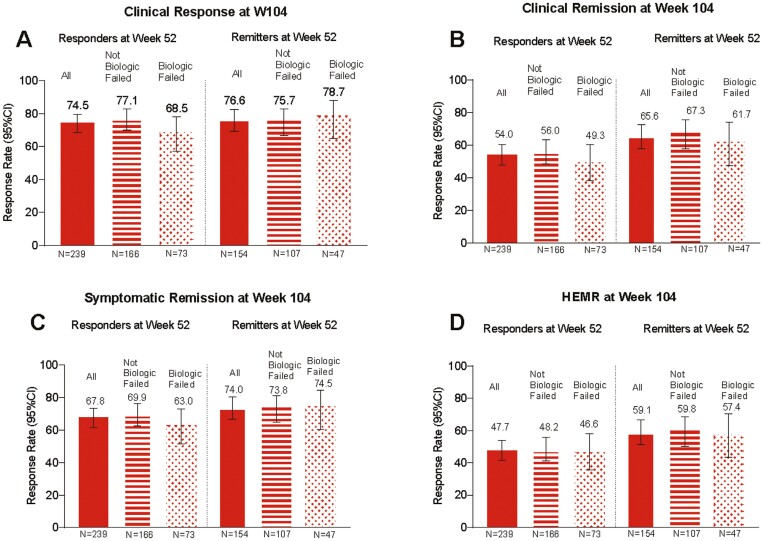
LUCENT-3 rates at 104 weeks of continuous treatment in LUCENT-2 week 52 responders and remitters by biologic-failed and not-biologic-failed treatment status for (A) clinical response, (B) clinical remission, (C) symptomatic remission, and (D) histologic-endoscopic mucosal remission (HEMR) (Geboes ≤2B.0 + endoscopic subscore [ES] = 0 or 1 [excluding friability]), nonresponder imputation. The modified intention-to-treat population was used with nonresponder imputation methods for missing data. Responders: ≥30% and 2-point decrease from baseline in the composite clinical endpoint of the sum of ES and stool frequency (SF) and rectal bleeding (RB) subscores, and RB = 0 or 1, or ≥1-point decrease from baseline. Remitters: modified Mayo score SF = 0 or SF = 1 with ≥1-point decrease from baseline; RB = 0; ES = 0 or 1. Biologic failed refers to those biologic-failed patients at LUCENT-1 induction baseline: prior inadequate response, loss of response, or intolerance to biologic therapy or Janus kinase inhibitors (tofacitinib). Not biologic failed refers to not-biologic-failed patients at LUCENT-1 induction baseline: patients not meeting biologic-failed definition. Symptomatic remission: SF = 0 or SF = 1 with ≥1-point decrease in modified Mayo score from baseline; RB = 0. CI, confidence interval.

Using nonresponder imputation, 74.5% of week 52 mirikizumab responders (n = 239) demonstrated clinical response at week 104 (Figure 2). Supplementary Table 2 and Supplementary Figure 1 provide additional data for week 52 mirikizumab responders and data for week 52 remitters (n = 154). Remission rates at week 104 for week 52 clinical responders were 54.0% clinical, 52.7% corticosteroid-free, 65.3% endoscopic, 47.7% HEMR, 67.8% symptomatic, and 50.2% bowel urgency (Figures 2 and 3). Week 52 responders achieving HEMI and bowel urgency clinically meaningful improvement at week 104 were 53.1% and 67.0%, respectively (Figure 3). For corticosteroid-free analyses, 33.1% of induction responders were on corticosteroids at baseline; 21.8% were on immunomodulators. Biologic-failed and not-biologic-failed subgroup data were generally similar, with differences of 0.7% to 13.0% depending on the endpoint (Figure 4, Supplementary Figure 1 and Supplementary Table 2).

**Figure 5.**
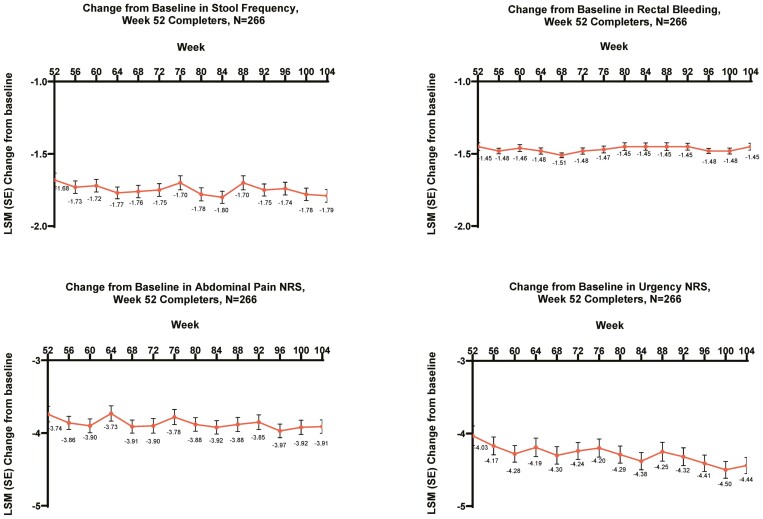
LUCENT-3 change from induction baseline in ulcerative colitis symptoms from weeks 52 through 104 (LUCENT-3 weeks 0-52) of continuous mirikizumab treatment in LUCENT-2 week 52 completers for (A) stool frequency (change in stool frequency modified Mayo score subscore from induction baseline), (B) rectal bleeding (change in rectal bleeding modified Mayo score subscore from induction baseline), (C) Abdominal Pain Numeric Rating Scale (NRS), and (D) Urgency NRS, mixed models for repeated measures. The modified intention-to-treat population was used with mixed models for repeated measures to estimate the mean change from baseline. The Abdominal Pain NRS measures change in abdominal pain score from induction baseline. The Urgency NRS measures change in bowel Urgency NRS score from induction baseline. LSM = least-squares mean.

**Figure 6.**
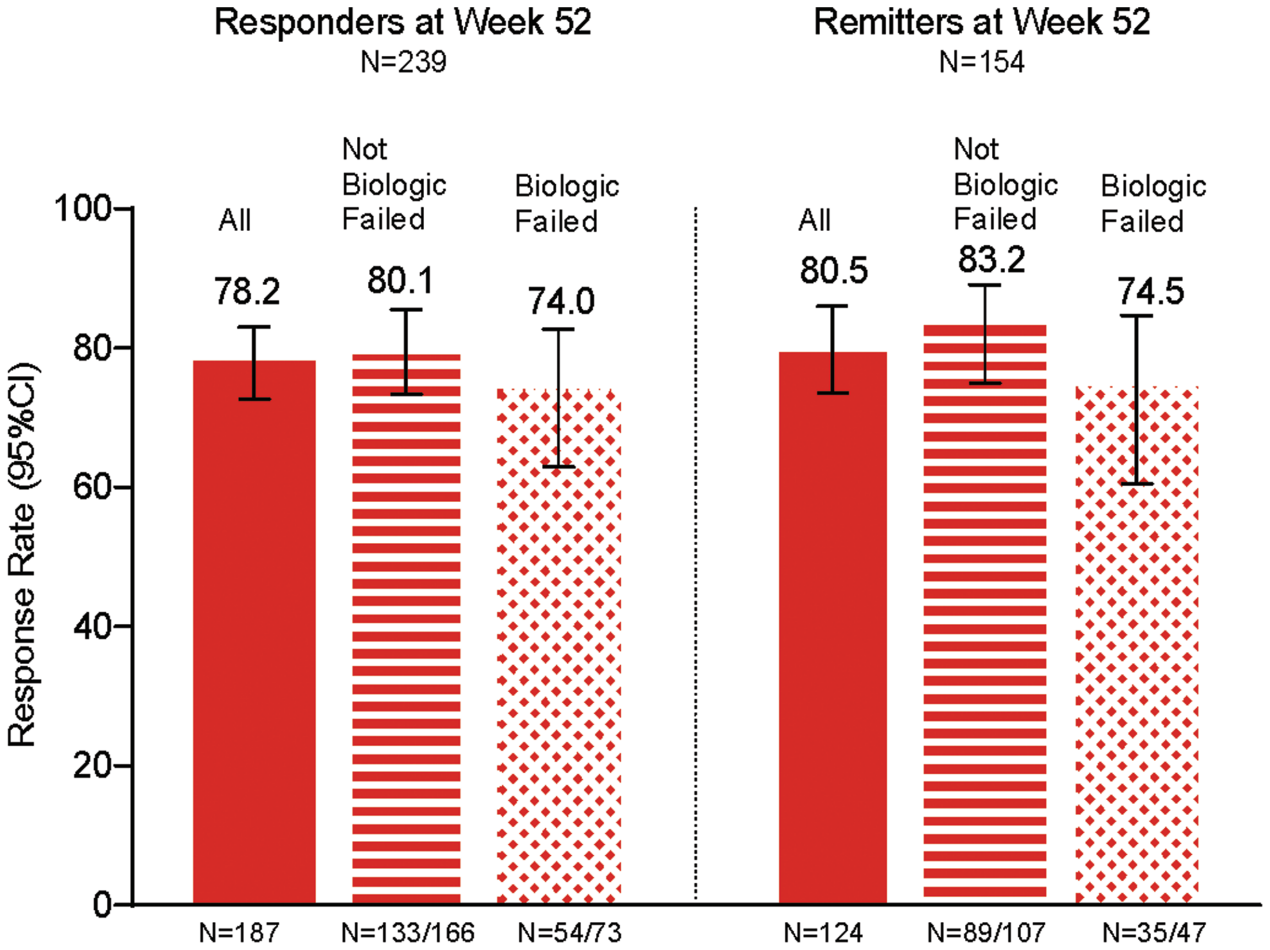
LUCENT-3 rate at 104 weeks of continuous treatment in LUCENT-2 week 52 responders and remitters by biologic-failed and not-biologic-failed treatment status for Inflammatory Bowel Disease Questionnaire (IBDQ) remission (IBDQ total score ≥170), nonresponder imputation. The modified intention-to-treat population was used with modified baseline observation carried forward and nonresponder imputation methods for missing data for continuous and categorical endpoints, respectively. Baseline was defined as the last nonmissing assessment recorded on or prior to the date of the first study drug administration at week 0 in the LUCENT-1 induction study. Inflammatory Bowel Disease Questionnaire remission rates are based on IBDQ total score ≥170; categorical data. Responders: ≥30% and 2-point decrease from baseline in the composite clinical endpoint of the sum of endoscopic subscore and stool frequency (SF) and rectal bleeding (RB) subscores, and RB = 0 or 1, or ≥1-point decrease from baseline. Remitters: modified Mayo score SF = 0 or SF = 1 with ≥1-point decrease from baseline; RB = 0; endoscopic subscore = 0 or 1. Biologic failed refers to biologic-failed patients at LUCENT-1 induction baseline: prior inadequate response, loss of response, or intolerance to biologic therapy or Janus kinase inhibitors (tofacitinib). Not biologic failed refers to not-biologic-failed patients at LUCENT-1 induction baseline: patients not meeting the biologic-failed definition. CI, confidence interval.

Using modified nonresponder imputation for week 52 mirikizumab responders and remitters provided categorical endpoint outcomes (Supplementary Table 3). These analyses included percent of patients categorized as nonresponders due to treatment discontinuation and percent of patients kept in analyses using multiple imputation who had sporadic missing data. Among week 52 mirikizumab responders, 87.2% demonstrated clinical response, and 62.8% demonstrated clinical remission at week 104. For week 52 mirikizumab remitters, 89.0% demonstrated clinical response, and 76.1% demonstrated clinical remission at week 104. Supplementary Table 3 provides response and remission rates across all other assessed outcomes. Figures 2 and 3 and Supplementary Figure 2 show efficacy endpoint data for week 52 mirikizumab responders and remitters, providing endpoint outcomes for nonresponder imputation, modified nonresponder imputation, and observed case analyses.

#### Maintenance of outcomes

For maintenance outcome analyses, only the patients who met the endpoint at week 52 (week 40 LUCENT-2) were included in the analyses to ascertain durable maintenance of that endpoint from 52 to 104 weeks of treatment. Figure 2 shows durable maintenance at week 104 (LUCENT-3) for clinical response (responders at week 52) and clinical remission (remitters at week 52) using nonresponder imputation, modified nonresponder imputation, and observed case methods. Using nonresponder imputation, 74.5% of patients maintained clinical response, and 65.6% maintained clinical remission. Supplementary Figure 3 shows 70.6% of patients maintained symptomatic response, 59.2% maintained HEMR, and 67.6% maintained bowel urgency remission. For modified nonresponder imputation at week 104 (LUCENT-3), 87.2% of patients maintained clinical response and 76.1% maintained clinical remission. Biologic-failed and not-biologic-failed patients had similar results to the overall population as shown in Supplementary Figure 4, which shows nonresponder imputation data.

#### Symptom scores over time

Patients treated with mirikizumab for 52 weeks (LUCENT-2, week 40) who continued mirikizumab treatment in LUCENT-3 for an additional 52 weeks demonstrated sustained symptom score reduction for stool frequency, rectal bleeding, abdominal pain, and bowel urgency from induction baseline (LUCENT-1, week 0) through week 104 of LUCENT-3 (Figure 5).

Using nonresponder imputation in the modified intention-to-treat population with a 95% CI, a ≥30% improvement in abdominal pain at week 104 (LUCENT-3 week 52) was observed in 168 of 224 (75.0% [95% CI, 68.9%-80.2%]) of induction responders with an Abdominal Pain Numeric Rating Scale ≥3 at induction baseline. Similar results were observed for maintenance responders, with 157 of 207 (75.8% [95% CI, 69.6%-81.2%]) showing improvement, regardless of biologic failure status. Biologic-failed maintenance responders were 39 of 61 (63.9% [95% CI, 51.4%-74.8%]); not-biologic-failed maintenance responders were 105 of 146 (71.9% [95% CI, 64.1%-78.6%]). For observed case analyses, in induction responders, a ≥30% improvement in abdominal pain was observed in 168 of 175 (96.0% [95% CI, 92.0%-98.0]). Similar results were observed for observed case in maintenance responders, with 157 of 162 (96.9% [95% CI, 93.0%-98.7%]) showing improvement.

#### Outcomes with extended induction

Using nonresponder imputation, among extended induction responders at week 52 (n = 81) (LUCENT-2 week 40), remission rates at week 104 were 34.6% clinical, 45.7% endoscopic, 33.3% HEMR, and 45.7% bowel urgency (Supplementary Figures 5-7). Patients achieving HEMI and bowel urgency clinically meaningful improvement were 40.7% and 59.7%, respectively. Biologic-failed and not-biologic-failed subgroup data for extended induction were generally similar to the overall extended induction group. Supplementary Table 4 provides modified nonresponder imputation and observed case clinical remission, clinical response, and symptomatic response data.

#### Outcomes with reinduction due to loss of response

There was a very low loss of response to mirikizumab during LUCENT-2. Out of 365 mirikizumab induction responders, only 19 patients experienced loss of response and received reinduction. Of these, 15 patients completed the rescue; 2 patients did not complete the rescue due to lack of efficacy, 1 due to withdrawal by patient and 1 due to pregnancy. Four patients were not included in the current interim analyses due to a site delay in protocol amendment approval, leaving 11 reinduction responders in the current analyses. For the 11 patients who completed rescue, were enrolled in LUCENT-3, and were eligible to be included in the interim analyses, 2 discontinued early (1 for an adverse event and 1 for lack of efficacy); the remaining patients continued because the study physician felt the patients were benefiting from the treatment even if they did not meet the study definition of clinical response. Of these, 54.5% (n = 6 of 11) attained clinical response and symptomatic remission at week 104 (week 52 LUCENT-3), while 9.1% (n = 1 of 11) attained clinical and alternate clinical remission, corticosteroid-free remission, HEMI, and HEMR at week 104. Additionally, 18.2% (n = 2 of 11) achieved endoscopic remission and 36.4% (n = 4 of 11) achieved bowel urgency remission. Eight (72.7%) patients achieved bowel urgency clinically meaningful improvement. At week 104, there was an additional 0.76 decrease in Urgency Numeric Rating Scale score from week 52 (LUCENT-3 week 0): week 52 least-squares mean: −3.72 (SE = 1.808); week 104 least-squares mean: −4.48 (SE = 0.850).

#### Quality-of-life outcomes

Using nonresponder imputation, the 239 patients with clinical response and 154 patients with clinical remission at week 52 (LUCENT-2 week 40) who entered the LUCENT-3 study were assessed for health-related quality of life. For these patients, least-squares mean improvements from the induction baseline in IBDQ total and domain scores were sustained at week 104, with LUCENT-2 clinical responders or remitters achieving over 60 points of IBDQ total score improvement (Supplementary Figure 8). Improvements in IBDQ scores were seen across all IBDQ domains: bowel symptoms, emotional function, social function, and systemic symptoms. IBDQ response at week 104 was seen in over 80% of patients and was comparable across biologic failure status subgroups. IBDQ remission rates in clinical responders (78.2%) and clinical remitters (80.5%) were sustained in patients regardless of whether they were in the biologic-failed or not-biologic-failed subgroups (Figure 6).

Using observed case analyses, 194 of 209 (92.8% [95% CI, 88.5%-95.6%]) patients with clinical response at week 52 achieved IBDQ response at week 104, and 187 of 210 (89.0% [95% CI, 84.1%-92.6%]) patients achieved IBDQ remission; 128 of 135 (94.8% [95% CI, 89.7%-97.5%]) patients with clinical remission at week 52 achieved IBDQ response at week 104 and 124 of 136 (91.2% [95% CI, 85.2%-94.9%]) patients achieved IBDQ remission.

For the extended induction population, using nonresponder imputation, 83 of 102 (81.4% [95% CI, 72.7%-87.7%]) achieved IBDQ response, and 78 of 102 (76.5% [95% CI, 67.4%-83.6%]) achieved IBDQ remission. Using observed case, 83 of 89 (93.3% [95% CI, 86.1%-96.9%]) achieved IBDQ response and 78 of 91 (85.7% [95% CI, 77.1%-91.5%]) achieved IBDQ remission.

### Safety

#### Adverse events

For the 52-week period of LUCENT-3 (week 0 to week 52 LUCENT-3; week 52 to week 104 continuous mirikizumab treatment), Table 1 provides the incidence of treatment-emergent adverse events for the overall induction responder safety population (see Patient Groups for population definitions). Severe treatment-emergent adverse events were reported in 4.5% of patients; 5.2% experienced serious adverse events, with 2.8% discontinuing treatment due to an adverse event. The most common treatment-emergent adverse events were pyrexia, diarrhea, injection site pain, abdominal pain, and gastroenteritis. There were no deaths through week 104.

**Table 1.** LUCENT-3 adverse events.

Outcome	200 mg mirikizumab Q4W SC (n = 289)^a^
**TEAEs** ^b^	184 (63.7)
Mild	99 (34.3)
Moderate	72 (24.9)
Severe	13 (4.5)
**SAEs**	15 (5.2)
**Most common TEAEs** ^c^	
COVID-19	35 (12.1)
Colitis ulcerative	22 (7.6)
Arthralgia	18 (6.2)
Headache	18 (6.2)
Nasopharyngitis	17 (5.9)
Pyrexia	13 (4.5)
Diarrhea	10 (3.5)
Injection site pain	10 (3.5)
Abdominal pain	9 (3.1)
Gastroenteritis	9 (3.1)
**AEs of special interest**	
Infections: all	87 (30.1)
Infections: serious	3 (1.0)
Infections: opportunistic^d^	5 (1.7)
Cerebrocardiovascular events^e^	2 (0.7)
Malignancies	0 (0)
Depression	1 (0.3)
Suicide/self-injury^f^	1 (0.3)
Hepatic	6 (2.1)
Immediate hypersensitivity reactions^g^	4 (1.4)
Injection site reactions^h^	16 (5.5)
**Death**	0 (0)
**Discontinuation due to AE** ^i^	8 (2.8)

Values are n (%).

Abbreviations: AE, adverse event; Q4W, every 4 weeks; SAE, serious adverse event; SC, subcutaneous; TEAE, treatment-emergent adverse event.

^a^The safety population was used for AE assessments and includes participants from Poland and Turkey affected by the electronic clinical outcome assessment error in LUCENT-1 and LUCENT-2, as well as patients on blinded mirikizumab at the end of LUCENT-2 who were not in remission or response and who are not included in the efficacy analysis.

^b^Patients with multiple occurrences of the same event are counted under the highest severity.

^c^Affecting ≥3% of patients.

^d^Narrow, 5 (1.7%). breakdown: herpes zoster, 3 (1.0%); esophageal candidiasis, 1 (0.3%); oral candidiasis, 1 (0.3%).

^e^Major adverse cardiac event, 1 (0.3%), determined by the investigator as not related to mirikizumab.

^f^Suicide attempt, determined by the investigator as not related to mirikizumab.

^g^Hypersensitivity reactions (narrow) included allergic sinusitis, 1 (0.3%); eczema, 1 (0.3%); injection site hypersensitivity, 1 (0.3%); and injection site urticaria, 1 (0.3%).

^h^Injection site pain, 10 (3.5%); injection site reaction, 5 (1.7%); injection site erythema, 3 (1.0%); injection site hypersensitivity, 1 (0.3%); injection site pruritus, 1 (0.3%); injection site urticaria, 1 (0.3%).

^i^Reasons: 1 dermatitis, 5 ulcerative colitis, 1 hematochezia, 1 meningitis.

For the 52-week period of LUCENT-3 in the induction responder safety population, 1 (0.4%) patient had elevated alanine aminotransferase ≥3× the upper limit of normal, 1 (0.4%) patient had elevated aspartate transaminase (≥3× the upper limit of normal [same patient as one with elevated alanine aminotransferase]), and 2 (0.7%) patients had elevated bilirubin (≥2× upper limit of normal). No patients had liver enzymes that were 5× or 10× the 2× upper limit of normal, nor did any patients meet Hy’s law criteria.^18^

## Discussion

With newer advanced therapeutics available for UC, treatment targets now include goals beyond endoscopic improvement, control of stool frequency, and control of rectal bleeding, including improvement of bowel urgency^19^ and histological inflammation.^20-22^ Moreover, treatment care gaps for UC exist for many patients in which treatment efficacy is not maintained over time, with approximately 40% of patients who respond to treatment subsequently losing response.^23^ LUCENT-3 is one of the first long-term extension studies in UC to provide data for endpoints such as clinical response, clinical remission, HEMI, HEMR, and endoscopic remission. Studies of other therapeutics generally only include symptom data.

Current data suggest mirikizumab provides long-term (104 weeks) sustained and durable efficacy for the majority of patients who initially respond to treatment, providing long-term efficacy across all studied endpoints while having a positive benefit-risk safety profile. Findings were consistent across clinical response/remission, endoscopic, histologic, symptomatic, patient-reported, and quality-of-life outcomes over 2 years of treatment, suggesting that mirikizumab treatment is effective at improving patient SF, RB, macroscopic and microscopic mucosal healing, bowel urgency, and abdominal pain that results in improved patient quality of life. Importantly, long-term efficacy was generally similar between those who had previously failed advanced therapies, those who had not failed advanced therapies, and the overall patient population. Moreover, mirikizumab provides efficacy for extended induction responders whose disease characteristics suggest that they had more severe disease at baseline than the induction responders, as well as reinduction responders.

Importantly, there are no head-to-head studies between IL-23 p19 inhibitors and other advanced therapies. Any indirect comparisons made between studies should be interpreted with caution due to differences in study designs, populations, levels of missing data, and statistical analysis methods. Although vedolizumab, an α4β7-integrin inhibitor approved for treatment of UC,^24^ and ustekinumab, an IL-12 and IL-23p40 inhibitor approved for the treatment of UC,^25^ have published long-term efficacy and safety data, no endoscopy data were obtained in those long-term extension studies. Conversely, the mirikizumab clinical development program included endoscopy, and histology was assessed in the LUCENT-3 extension study, allowing for endoscopy to be used in response and remission definitions, and HEMI and HEMR endpoints to be analyzed. The current HEMR definition used included absence of mucosal neutrophils that accumulate with persistent acute UC inflammation,^26^ which is important, as the absence of intraepithelial neutrophils is recommended as a requirement for defining histological remission.^26-28^ Mirikizumab demonstrated it can deliver early resolution of histological and endoscopic inflammation, which is associated with better UC outcomes.^29^ The current data demonstrate durable long-term histologic-endoscopic efficacy.

Bowel urgency was assessed with the Urgency Numeric Rating Scale, a validated scale that enables assessment of bowel urgency severity improvement over time rather than the simple yes vs no assessment which has historically been used.^14,30,31^ This is important because many patients with UC consider controlling bowel urgency more important than RB or SF.^32,33^ Mirikizumab demonstrated that it can deliver early resolution of bowel urgency that is associated with better UC outcomes.^29^ The current data demonstrate sustained long-term bowel urgency efficacy.

Current first-line treatment recommendations for moderate-to-severe UC are commonly anti-tumor necrosis factor α biologics such as adalimumab or infliximab^34^; however, some primary responders experience secondary loss of response.^35^ Mirikizumab demonstrated maintained long-term efficacy for induction responders. This is likely due to the ability of mirikizumab to decrease the expression of transcripts associated with resistance to anti-tumor necrosis factor therapy in cell types thought to be instrumentally involved.^23,36,37^ Mirikizumab induces differentially expressed gene transcript changes that are sustained through week 52 and are associated with a distinct molecular healing pathway.^36^ This is important, as molecular healing is currently considered a potential UC treat-to-target goal.^37^ Mirikizumab may therefore benefit a subset of patients with UC who do not maintain response with other treatments.

Safety findings were consistent with findings from LUCENT-1 and LUCENT-2 studies, in which it was observed that overall adverse events were numerically greater for placebo than mirikizumab-treated patients.^10^ During LUCENT-1 (induction) and LUCENT-2 (maintenance), the incidence of hepatic enzyme elevations was low but was more frequent in mirikizumab-treated patients.^10^ The current data suggest that it is unlikely that there are any clinically meaningful long-term hepatic enzyme elevations in patients treated with mirikizumab for 104 weeks. Infection and cancer rates do not indicate a profound systemic immune suppression. COVID-19 (12.1%) was observed as a most common adverse event (≥3%), which is not surprising, as this study was held during the COVID-19 epidemic. Of the 35 COVID-19 cases, only 1 was considered serious and for that patient the COVID-19 resolved without dose change. UC as a most common adverse event (7.6%) is linked to a worsening or re-emergence of symptoms of the disease being studied and is not surprising because UC can be associated with flares. Of note, related to adverse events of special interest UC treatments, no malignancies were observed.

Future analyses from the ongoing open-label extension LUCENT-3 (NCT03519945) study will provide robust long-term 3- and 4-year data.

### Limitations

LUCENT-3 was an open-label study with no comparator. Based on the complex study design and patient flow across LUCENT-1/2/3, and the current focus on investigating durability and sustainability of efficacy among mirikizumab responders, the current analyses do not examine the full LUCENT program population. Induction placebo responders and nonresponders were not included, and mirikizumab patients who did not respond to extended induction or reinduction did not move forward into LUCENT-3. This affected the overall denominator in the percentage of patients meeting endpoint calculations (eg, remission). As with other biologic treatments studied for UC, the clinical remission rate reported can be misinterpreted based on the sample composition. This is because these trials use a responder methodology, in which only patients who responded during induction are rerandomized to maintenance. The net clinical remission rate defined as the actual percentage of patients enrolled during induction who are in remission at the end of long-term maintenance is inherently lower than that of the responder population.

## Conclusions

These data support the long-term benefit of continuous mirikizumab treatment for 104 weeks, with or without extended induction, on clinical, endoscopic, histologic, and symptomatic endpoints, including for biologic-failed patients. No new safety signals were identified, and the discontinuation rate due to adverse events was 2.8%.

## Supplementary data

Supplementary data is available at *Inflammatory Bowel Diseases* online.

izae024_suppl_Supplementary_Material

## References

[1] Irvine EJ. Quality of life of patients with ulcerative colitis: past, present, and future. Inflamm Bowel Dis. 2008;14(4):554-565. 17973299 10.1002/ibd.20301

[2] Dubinsky M , Panaccione R, Lopez-Sanroman A, et al P024 Patient and healthcare provider views on ulcerative colitis treatment goals and quality of life: results of a global ulcerative colitis narrative survey. Gastroenterology. 2019;156(3):S17-S18.

[3] Le Berre C , Honap S, Peyrin-Biroulet L. Ulcerative colitis. Lancet. 2023;402(10401):571-584. 37573077 10.1016/S0140-6736(23)00966-2

[4] Daperno M , Armuzzi A, Danese S, et al Unmet medical needs in the management of ulcerative colitis: results of an Italian Delphi consensus. Gastroenterol Res Pract. 2019;2019:3108025. 10.1155/2019/3108025 PMC6745180 31565051

[5] Danese S , Allez M, van Bodegraven AA, et al Unmet medical needs in ulcerative colitis: an expert group consensus. Dig Dis. 2019;37(4):266-283. 30726845 10.1159/000496739

[6] Gordon JP , McEwan PC, Maguire A, Sugrue DM, Puelles J. Characterizing unmet medical need and the potential role of new biologic treatment options in patients with ulcerative colitis and Crohn’s disease: a systematic review and clinician surveys. Eur J Gastroenterol Hepatol. 2015;27(7):804-812. 25933126 10.1097/MEG.0000000000000378 PMC4892747

[7] Kobayashi T , Siegmund B, Le Berre C, et al Ulcerative colitis. Nat Rev Dis Primers. 2020;6(1):74. 32913180 10.1038/s41572-020-0205-x

[8] Abraham C , Cho JH. IL-23 and autoimmunity: new insights into the pathogenesis of inflammatory bowel disease. Annu Rev Med. 2009;60:97-110. 18976050 10.1146/annurev.med.60.051407.123757

[9] Kobayashi T , Okamoto S, Hisamatsu T, et al IL23 differentially regulates the Th1/Th17 balance in ulcerative colitis and Crohn’s disease. Gut. 2008;57(12):1682-1689. 18653729 10.1136/gut.2007.135053

[10] D’Haens G , Dubinsky M, Kobayashi T, et al; LUCENT Study Group. Mirikizumab as induction and maintenance therapy for ulcerative colitis. N Engl J Med. 2023;388(26):2444-2455. 37379135 10.1056/NEJMoa2207940

[11] Sandborn WJ , Ferrante M, Bhandari BR, et al Efficacy and safety of mirikizumab in a randomized phase 2 study of patients with ulcerative colitis. Gastroenterology. 2020;158(3):537-549.e10. 31493397 10.1053/j.gastro.2019.08.043

[12] Sandborn WJ , Ferrante M, Bhandari BR, et al Efficacy and safety of continued treatment with mirikizumab in a phase 2 trial of patients with ulcerative colitis. Clin Gastroenterol Hepatol. 2022;20(1):105-115.e14. 32950748 10.1016/j.cgh.2020.09.028

[13] Public Policy Committee, International Society of Pharmacoepidemiology. Guidelines for good pharmacoepidemiology practice (GPP). Pharmacoepidemiol Drug Saf. 2016;25(1):2-10. 26537534 10.1002/pds.3891

[14] Dubinsky MC , Shan M, Delbecque L, et al Psychometric evaluation of the Urgency NRS as a new patient-reported outcome measure for patients with ulcerative colitis. J Patient Rep Outcomes. 2022;6(1):114. 36334163 10.1186/s41687-022-00522-2 PMC9637076

[15] Wilson EB. Probable inference, the law of succession, and statistical inference. J Am Stat Assoc. 1927;22(158):209-212.

[16] Newcombe RG. Interval estimation for the difference between independent proportions: comparison of eleven methods. Stat Med. 1998;17(8):873-890. 9595617 10.1002/(sici)1097-0258(19980430)17:8<873::aid-sim779>3.0.co;2-i

[17] Rubin DB. Multiple Imputation for Nonresponse in Surveys. 1st ed. Wiley; 1987.

[18] Robles–Diaz M , Lucena MI, Kaplowitz N. Use of Hy’s Law and a new composite algorithm to predict acute liver failure in patients with drug-induced liver injury. Gastroenterology. 2014;147(1):109-118.e5. 24704526 10.1053/j.gastro.2014.03.050

[19] Dubinsky MC , Clemow DB, Hunter-Gibble T, et al Clinical effect of mirikizumab treatment on bowel urgency in patients with moderately to severely active ulcerative colitis and the clinical relevance of bowel urgency improvement for disease remission. Crohns Colitis 360. 2022;5(1):otac044. 36777368 10.1093/crocol/otac044 PMC9802448

[20] Rubin DT , Ananthakrishnan AN, Siegel CA, Sauer BG, Long MD. ACG clinical guideline: ulcerative colitis in adults. Am J Gastroenterol. 2019;114(3):384-413. 30840605 10.14309/ajg.0000000000000152

[21] Danese S , Roda G, Peyrin-Biroulet L. Evolving therapeutic goals in ulcerative colitis: towards disease clearance. Nat Rev Gastroenterol Hepatol. 2020;17(1):1-2. 31520081 10.1038/s41575-019-0211-1

[22] Turner D , Ricciuto A, Lewis A, et al; International Organization for the Study of IBD. STRIDE-II: an update on the selecting therapeutic targets in inflammatory bowel disease (STRIDE) initiative of the International Organization for the Study of IBD (IOIBD): determining therapeutic goals for treat-to-target strategies in IBD. Gastroenterology. 2021;160(5):1570-1583. 33359090 10.1053/j.gastro.2020.12.031

[23] Steere B , Schmitz J, Powell N, et al Mirikizumab regulates genes involved in ulcerative colitis disease activity and anti-TNF resistance: results from a phase 2 study. Clin Transl Gastroenterol. 2023;14(7):e00578. 36881820 10.14309/ctg.0000000000000578 PMC10371316

[24] Loftus EV , Colombel JF, Feagan BG, et al Long-term efficacy of vedolizumab for ulcerative colitis. J Crohns Colitis. 2017;11(4):400-411. 27683800 10.1093/ecco-jcc/jjw177

[25] Panaccione R , Danese S, Sandborn WJ, et al Ustekinumab is effective and safe for ulcerative colitis through 2 years of maintenance therapy. Aliment Pharmacol Ther. 2020;52(11-12):1658-1675. 33086438 10.1111/apt.16119 PMC8776399

[26] Fournier BM , Parkos CA. The role of neutrophils during intestinal inflammation. Mucosal Immunol. 2012;5(4):354-366. 22491176 10.1038/mi.2012.24

[27] Pai RK , Hartman DJ, Rivers CR, et al Complete resolution of mucosal neutrophils associates with improved long-term clinical outcomes of patients with ulcerative colitis. Clin Gastroenterol Hepatol. 2020;18(11):2510-2517.e5. 31843598 10.1016/j.cgh.2019.12.011

[28] Magro F , Doherty G, Peyrin-Biroulet L, et al ECCO Position Paper: harmonization of the approach to ulcerative colitis histopathology. J Crohns Colitis. 2020;14(11):1503-1511. 32504534 10.1093/ecco-jcc/jjaa110

[29] Magro F , Pai RK, Kobayashi T, et al Resolving histological inflammation in ulcerative colitis with mirikizumab in the LUCENT induction and maintenance trial programs. J Crohns Colitis. 2023;17(9):1457-1470. 37057827 10.1093/ecco-jcc/jjad050 PMC10588772

[30] Dubinsky MC , Irving PM, Panaccione R, et al Incorporating patient experience into drug development for ulcerative colitis: development of the Urgency Numeric Rating Scale, a patient-reported outcome measure to assess bowel urgency in adults. J Patient Rep Outcomes. 2022;6(1):31. 35362902 10.1186/s41687-022-00439-w PMC8975984

[31] Dubinsky MC , Newton L, Delbecque L, et al Exploring disease remission and bowel urgency severity among adults with moderate to severe ulcerative colitis: a qualitative study. Patient Relat Outcome Meas. 2022;13:287-300. 36582542 10.2147/PROM.S378759 PMC9793422

[32] Louis E , Ramos-Goñi JM, Cuervo J, et al A qualitative research for defining meaningful attributes for the treatment of inflammatory bowel disease from the patient perspective. Patient. 2020;13(3):317-325. 31997116 10.1007/s40271-019-00407-5 PMC7210247

[33] van Deen WK , Obremskey A, Moore G, et al An assessment of symptom burden in inflammatory bowel diseases to develop a patient preference-weighted symptom score. Qual Life Res. 2020;29(12):3387-3396. 32813264 10.1007/s11136-020-02606-2

[34] Feuerstein JD , Isaacs KL, Schneider Y, et al; AGA Institute Clinical Guidelines Committee. AGA clinical practice guidelines on the management of moderate to severe ulcerative colitis. Gastroenterology. 2020;158(5):1450-1461. 31945371 10.1053/j.gastro.2020.01.006 PMC7175923

[35] Gisbert JP , Chaparro M. Primary failure to an anti-TNF agent in inflammatory bowel disease: switch (to a second anti-TNF agent) or swap (for another mechanism of action)? J Clin Med. 2021;10(22):5318. 34830595 10.3390/jcm10225318 PMC8625924

[36] Johnson T , Steere B, Zhang P, et al DOP09 Mirikizumab-induced transcriptome changes in patient biopsies at week 12 are maintained through week 52 in patients with ulcerative colitis. J Crohns Colitis. 2021;15(Suppl_1):S047-S048. 10.14309/ctg.0000000000000630 PMC10684203 37594044

[37] Ungaro R , Colombel JF, Lissoos T, Peyrin-Biroulet L. A treat-to-target update in ulcerative colitis: a systematic review. Am J Gastroenterol. 2019;114(6):874-883. 30908297 10.14309/ajg.0000000000000183 PMC6553548

