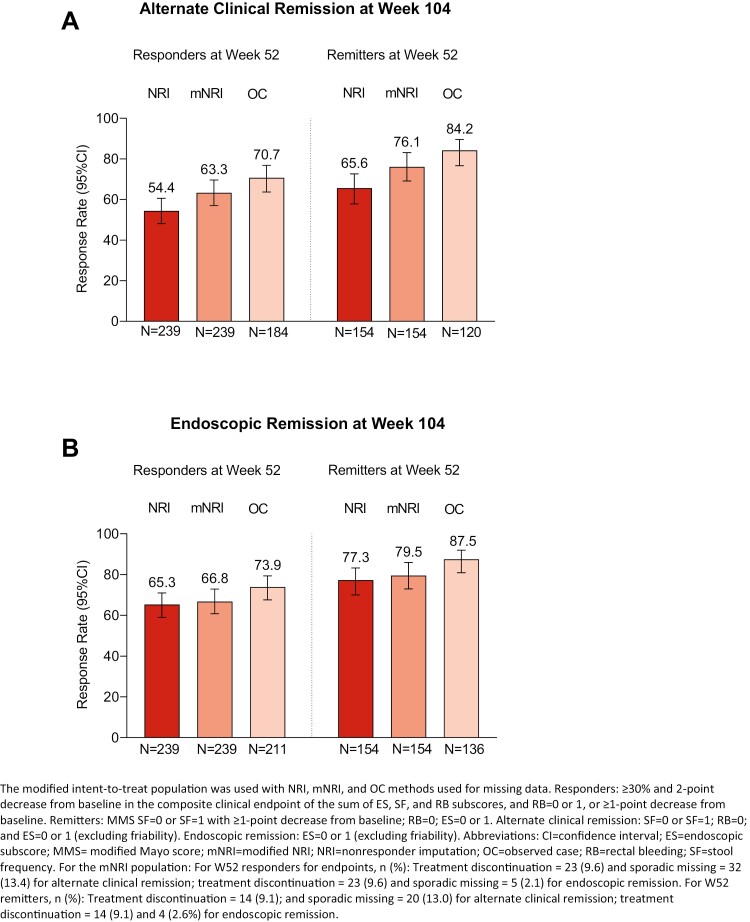# Correction to: Two-Year Efficacy and Safety of Mirikizumab Following 104 Weeks of Continuous Treatment for Ulcerative Colitis: Results From the LUCENT-3 Open-Label Extension Study

**DOI:** 10.1093/ibd/izae096

**Published:** 2024-04-27

This is a **correction** to:

Bruce E Sands, Geert D’Haens, David B Clemow, Peter M Irving, Jordan T Johns, Theresa Hunter Gibble, Maria T Abreu, Scott Lee, Tadakazu Hisamatsu, Taku Kobayashi, Marla C Dubinsky, Severine Vermeire, Corey A Siegel, Laurent Peyrin-Biroulet, Richard E Moses, Joe Milata, Vipin Arora, Remo Panaccione, Axel Dignass, Two-Year Efficacy and Safety of Mirikizumab Following 104 Weeks of Continuous Treatment for Ulcerative Colitis: Results From the LUCENT-3 Open-Label Extension Study, *Inflammatory Bowel Diseases*, 2024;, izae024, https://doi.org/10.1093/ibd/izae024

In the originally published version of this manuscript, there was an error in the results section of the abstract. The bolded numbers should be swapped in the following sentence:

From:

Among W52 mirikizumab responders, clinical response at W104 was 74.5%, 87.2%, and 96.7% and clinical remission was **76.6%, 89.0%, and 98.3%** for NRI, mNRI, and OC, respectively. Among W52 mirikizumab remitters, clinical response at W104 was **54.0%, 62.8%, and 70.1%** and clinical remission was 65.6%, 76.1%, and 84.2%.

To:

Among W52 mirikizumab responders, clinical response at W104 was 74.5%, 87.2%, and 96.7% and clinical remission was **54.0%, 62.8%, and 70.1%** for NRI, mNRI, and OC, respectively. Among W52 mirikizumab remitters, clinical response at W104 was **76.6%, 89.0%, and 98.3%** and clinical remission was 65.6%, 76.1%, and 84.2%.

Additionally,

The “N” values for Supplemental Figure 2, Panel B, was incorrect. The values should be as following:

Original: N=156 N=156 N=156 N=119 N=119 N=119

Corrected: N=239 N=239 N=211 N=154 N=154 N=136

These errors have been corrected.