# Supplementary material for: Two-Year Efficacy and Safety of Mirikizumab Following 104 Weeks of Continuous Treatment for Ulcerative Colitis: Results From the LUCENT-3 Open-Label Extension Study
Source: Inflamm Bowel Dis. 2024 Mar 9;30(12):2245–58. doi: 10.1093/ibd/izae024 (PMC11630283; doi:10.1093/ibd/izae024)
Supplement: izae024_suppl_Supplementary_Material [file izae024_suppl_supplementary_material.docx]

**Supplemental Figure Legends**

Supplemental Figure 1: LUCENT-3 Rates at 104 Weeks of Continuous Treatment in LUCENT-2 Week 52 Responders and Remitters by Biologic Failed and Not Failed Treatment Status for A: Alternate Clinical Remission, B: Corticosteroid-free Remission, C: Endoscopic Remission, D: HEMI, E: Bowel Urgency CMI, F: Bowel Urgency Remission, NRI

Supplemental Figure 2. LUCENT-3 Rates at 104 Weeks of Continuous Treatment in LUCENT-2 Week 52 Responders and Remitters for A: Alternative Clinical Remission, B: Endoscopic Remission, NRI, mNRI, OC

Supplemental Figure 3. LUCENT-3 Maintenance Rates at 104 Weeks of Continuous Treatment in LUCENT-2 Week 52 Endpoint Remitters for A: Symptomatic Remission, B: HEMR, C: Bowel Urgency Remission, NRI, mNRI, OC

Supplemental Figure 4. LUCENT-3 Maintenance Rates at 104 Weeks of Continuous Treatment in LUCENT-2 Week 52 Endpoint Responders and Remitters by Biologic Failed and Not Failed Treatment Status for A: Clinical Response, B: Clinical Remission, C: Symptomatic Remission, D: HEMR, E: Bowel Urgency Remission, NRI, mNRI, OC

Supplemental Figure 5. LUCENT-3 Extended Induction Rates at 104 Weeks of Continuous Treatment in LUCENT-2 Week 52 Responders by Biologic Failed and Not Failed Treatment Status for A: Clinical Remission, B: Corticosteroid-free Remission, C: Symptomatic Remission, NRI

Supplemental Figure 6. LUCENT-3 Extended Induction Rates at 104 Weeks of Continuous Treatment in LUCENT-2 Week 52 Responders by Biologic Failed and Not Failed Treatment Status for A: Endoscopic Remission, B: HEMI, C: HEMR, NRI

Supplemental Figure 7. LUCENT-3 Extended Induction Rates at 104 Weeks of Continuous Treatment in LUCENT-2 Week 52 Responders by Biologic Failed and Not Failed Treatment Status for A: Bowel Urgency CMI, B: Bowel Urgency Remission, NRI

Supplemental Figure 8. LUCENT-3 Rates at 104 Weeks of Continuous Treatment in LUCENT-2 Week 52 Responders and Remitters for A: IBDQ Change from Baseline, B: IBDQ Response (IBDQ Total Score ≥16-point Improvement), NRI

**Supplemental Table 1. Baseline Demographics and Patient Characteristics**

| **Attribute** | **Induction Responders**  **200 mg MIRI SC Q4W**  **(N = 266)** | **Extended Induction Responders**  **200mg MIRI SC Q4W**  **(N = 102)** |
| --- | --- | --- |
| **Age (years), mean (SD)** | 43.4 (13.98) | 45.9 (13.46) |
| **Male, n (%)** | 160 (60.2) | 58 (56.9) |
| **Weight (kg), mean (SD)** | 71.65 (17.215) | 71.57 (16.215) |
| **BMI category, n (%)** |  |  |
| Underweight (<18.5 kg/m^2^) | 17 (6.4) | 3 (2.9) |
| Normal (≥18.5 and <25 kg/m^2^) | 152 (57.1) | 56 (54.9) |
| Overweight (≥25 and <30 kg/m^2^) | 66 (24.8) | 27 (26.5) |
| Obese or extreme obese (≥30 kg/m^2^) | 31 (11.7) | 16 (15.7) |
| **Race, n (%)** |  |  |
| White | 183 (69.3) | 72 (70.6) |
| Black or African American | 4 (1.5) | 2 (2.0) |
| Asian | 77 (29.2) | 27 (26.5) |
| Other | 0 (0) | 1 (1.0) |
| **Geographic region, n (%)** |  |  |
| North America | 35 (13.2) | 11 (10.8) |
| Europe | 81 (30.5) | 31 (30.4) |
| Western Europe | 44 (16.5) | 15 (14.7) |
| Eastern Europe | 37 (13.9) | 16 (15.7) |
| Other | 150 (56.4) | 60 (58.8) |
| Asia | 74 (27.8) | 27 (26.5) |
| Central America/South America | 4 (1.5) | 2 (2.0) |
| Rest of the world | 72 (27.1) | 31 (30.4) |
| **Duration of ulcerative colitis (years), mean (SD)** | 6.62 (6.370) | 7.49 (7.190) |
| **Disease location, n (%)** |  |  |
| Proctitis | 1 (0.4) | 0 (0) |
| Left-side colitis | 178 (66.9) | 60 (58.8) |
| Pancolitis | 87 (32.7) | 42 (41.2) |
| **Baseline modified Mayo score category, n (%)** |  |  |
| Mild (1–3) | 0 (0) | 1 (1.0) |
| Moderate (4–6) | 131 (49.2) | 40 (39.2) |
| Severe (7–9) | 135 (50.8) | 61 (59.8) |
| **Total Mayo score, mean (SD)** | 8.8 (1.64) | 9.0 (1.55) |
| **Endoscopic Mayo subscore, n (%)** |  |  |
| Moderate (2) | 90 (33.8) | 26 (25.5) |
| Severe (3) | 176 (66.2) | 76 (74.5) |
| **Urgency NRS, mean (SD)** | 5.9 (2.21) | 6.3 (2.09) |
| **Fecal calprotectin (µg/g), median (range)** | 1482.0  (15, 31680) | 1538.5  (15, 22364) |
| **C-reactive protein (mg/L), median (range)** | 3.7 (0, 92) | 4.8 (0, 60) |
| **Baseline corticosteroid use, n (%)** | 88 (33.1) | 42 (41.2) |
| **Baseline immunomodulator use, n (%)** | 58 (21.8) | 27 (26.5) |
| **Prior biologic or tofacitinib failure, n (%)** | 85 (32.0) | 43 (42.2) |
| **Anti-TNF failure, n (%)** | 77 (28.9) | 40 (39.2) |
| **Vedolizumab failure, n (%)** | 27 (10.2) | 19 (18.6) |
| **Tofacitinib failure, n (%)** | 5 (1.9) | 4 (3.9) |
| **Number of failed biologics or tofacitinib, n (%)** |  |  |
| 0 | 181 (68.0) | 59 (57.8) |
| 1 | 50 (18.8) | 17 (16.7) |
| ≥2 | 35 (13.2) | 26 (25.5) |
| The modified intent-to-treat population was used. Abbreviations: BMI=body mass index; MIRI=mirikizumab; Q4W=every 4 weeks; NRS=numeric rating scale; SC=subcutaneous; SD=standard deviation; TNF=tumor necrosis factor. | | |

**Supplemental Table 2. LUCENT-3 Rates at 104 Weeks of Continuous Treatment in LUCENT-2 Week 52 Responders and Remitters by Biologic Failed and Not Failed Treatment Status, NRI**

|  | **In Week 52 Responders^a^** | | |  | **In Week 52 Remitters^b^** | | |
| --- | --- | --- | --- | --- | --- | --- | --- |
| **Endpoints at Week 104*** | **N = 239**  **% All^c^**  **(95% CI^d^)** | **N = 166**  **% Not Biologic Failed^e^**  **(95% CI^d^)** | **N = 73**  **% Biologic Failed^f^**  **(95% CI^d^)** |  | **N = 154**  **% All^c^**  **(95% CI^d^)** | **N = 107**  **% Not Biologic Failed^e^**  **(95% CI^d^)** | **N = 47**  **% Biologic Failed^f^**  **(95% CI^d^)** |
| Alternate clinical remission | 54.4  (48.1, 60.6) | 56.6  (49.0, 63.9) | 49.3  (38.2, 60.5) |  | 65.6  (57.8, 72.6) | 67.3  (57.9, 75.4) | 61.7  (47.4, 74.2) |
| Bowel urgency CMI | N = 224^g^ | N = 155^g^ | N = 69^g^ |  | N = 147^g^ | N = 102^g^ | N = 45^g^ |
|  | 67.0  (60.6, 72.8) | 71.0  (63.4, 77.5) | 58.0  (46.2, 68.9) |  | 67.3  (59.4, 74.4) | 68.6  (59.1, 76.8) | 64.4  (49.8, 76.8) |
| Bowel urgency remission | 50.2  (43.9, 56.5) | 54.2  (46.6, 61.6) | 41.1  (30.5, 52.6) |  | 51.3  (43.5, 59.1) | 55.1  (45.7, 64.2) | 42.6  (29.5, 56.7) |
| Clinical remission | 54.0  (47.6, 60.2) | 56.0  (48.4, 63.4) | 49.3  (38.2, 60.5) |  | 65.6  (57.8, 72.6) | 67.3  (57.9, 75.4) | 61.7  (47.4, 74.2) |
| Clinical response | 74.5  (68.6, 79.6) | 77.1  (70.1, 82.8) | 68.5  (57.1, 78.0) |  | 76.6  (69.3, 82.6) | 75.7  (66.8, 82.8) | 78.7  (65.1, 88.0) |
| CSF remission | 52.7  (46.4, 59.0) | 54.8  (47.2, 62.2) | 47.9  (36.9, 59.2) |  | 64.3  (56.5, 71.4) | 66.4  (57.0, 74.6) | 59.6  (45.3, 72.4) |
| Endoscopic remission | 65.3  (59.0, 71.0) | 68.7  (61.3, 75.2) | 57.5  (46.1, 68.2) |  | 77.3  (70.0, 83.2) | 80.4  (71.9, 86.8) | 70.2  (56.0, 81.3) |
| HEMI | 53.1  (46.8, 59.4) | 54.2  (46.6, 61.6) | 50.7  (39.5, 61.8) |  | 66.2  (58.5, 73.2) | 67.3  (57.9, 75.4) | 63.8  (49.5, 76.0) |
| HEMR | 47.7  (41.5, 54.0) | 48.2  (40.7, 55.7) | 46.6  (35.6, 57.9) |  | 59.1  (51.2, 66.5 | 59.8  (50.3, 68.6) | 57.4  (43.3, 70.5) |
| IBDQ remission | 78.2  (72.6, 83.0) | 80.1  (73.4, 85.5) | 74.0  62.9, 82.7 |  | 80.5  (73.6, 86.0) | 83.2  (75.0, 89.1) | 74.5  (60.5, 84.7) |
| IBDQ response | 81.2  (75.7, 85.6) | 81.3  (74.7, 86.5) | 80.8  (70.3, 88.2) |  | 83.1  (76.4, 88.2) | 82.2  (73.9, 88.3) | 85.1  (72.3, 92.6) |
| Symptomatic remission | 67.8  (61.6, 73.4) | 69.9  (62.5, 76.3) | 63.0  (51.5, 73.2) |  | 74.0  (66.6, 80.3) | 73.8  (64.8, 81.2) | 74.5  (60.5, 84.7) |
| Abbreviations: CI=confidence interval; CMI=clinically meaningful improvement; CSF=corticosteroid free; ES=endoscopic subscore; HEMI=histologic-endoscopic mucosal improvement; HEMR=histologic-endoscopic mucosal remission; IBDQ=Inflammatory Bowel Disease Questionnaire; MMS=modified Mayo score; NRI=nonresponder imputation; NRS=numeric rating scale; RB=rectal bleeding; SF=stool frequency.  * See manuscript section Methods – Outcome Measures for specific endpoint definitions.  ^a^ Responders: ≥30% and 2-point decrease from baseline in the composite clinical endpoint of the sum of ES, SF, and RB subscores, and RB=0 or 1, or ≥1 pt decrease from baseline.  ^b^ Remitters: MMS SF=0 or SF=1 with ≥1-point decrease from baseline; RB=0; ES=0 or 1.  ^c^ The modified intent-to-treat population was used with NRI methods for missing data.  ^d^ Response confidence intervals are constructed using Wilson method, without continuity correction.  ^e^ Not biologic failed: failed conventional treatments (i.e., immunomodulators/corticosteroids); may include some participants who were exposed to but did not fail biologic treatment.  ^f^ Biologic failed: Inadequate response, loss of response, or intolerant to a biologic therapy or the Janus kinase inhibitors for UC.  ^g^ In patients who had Urgency NRS ≥3 at induction baseline. | | | | | | | |

**Supplemental Table 3:**  **LUCENT-3 Rates at 104 Weeks of Continuous Treatment in LUCENT-2 Week 52 Responders and Remitters, mNRI**

| **Endpoint*** | **W52 Subgroup (N)** | **Treatment Discontinuation, n (%)** | **Sporadic Missing, n (****%)^a^** | **Response, %^b^** | **Response, 95% CI^c^** |
| --- | --- | --- | --- | --- | --- |
| Alternate clinical remission | Responder (239) | 23 (9.6) | 32 (13.4) | 63.3 | 57.0, 69.6 |
|  | Remitter (154) | 14 (9.1) | 20 (13.0) | 76.1 | 69.1, 83.1 |
| Clinical remission | Responder (239) | 23 (9.6) | 32 (13.4) | 62.8 | 56.4, 69.1 |
|  | Remitter (154) | 14 (9.1) | 20 (13.0) | 76.1 | 69.1, 83.1 |
| Clinical response | Responder (239) | 23 (9.6) | 32 (13.4) | 87.2 | 82.8, 91.6 |
|  | Remitter (154) | 14 (9.1) | 20 (13.0) | 89.0 | 83.8, 94.1 |
| CSF remission | Responder (239) | 23 (9.6) | 32 (13.4) | 61.4 | 55.0, 67.7 |
|  | Remitter (154) | 14 (9.1) | 20 (13.0) | 74.7 | 67.6, 81.9 |
| Symptomatic remission | Responder (239) | 23 (9.6) | 32 (13.4) | 79.2 | 73.8, 84.7 |
|  | Remitter (154) | 14 (9.1) | 20 (13.0) | 85.9 | 80.2, 91.7 |
| Endoscopic remission | Responder (239) | 23 (9.6) | 5 (2.1) | 66.8 | 60.8, 72.9 |
|  | Remitter (154) | 14 (9.1) | 4 (2.6) | 79.5 | 73.0, 85.9 |
| HEMI | Responder (239) | 23 (9.6) | 13 (5.4) | 57.1 | 50.8, 63.5 |
|  | Remitter (154) | 14 (9.1) | 9 (5.8) | 71.2 | 63.9, 78.5 |
| HEMR | Responder (239) | 23 (9.6) | 13 (5.4) | 51.6 | 45.1, 58.1 |
|  | Remitter (154) | 14 (9.1) | 9 (5.8) | 63.9 | 56.2, 71.6 |
| Bowel urgency CMI | Responder (224) | 22 (9.8) | 29 (12.9) | 77.8 | 72.1, 83.6 |
|  | Remitter (147) | 14 (9.5) | 18 (12.2) | 77.5 | 70.3, 84.7 |
| Bowel urgency Remission | Responder (239) | 23 (9.6) | 33 (13.8) | 58.6 | 51.7, 65.4 |
|  | Remitter (154) | 14 (9.1) | 20 (13.0) | 59.8 | 51.6, 68.0 |
| Abbreviations: CI = confidence interval; CMI = clinically meaningful improvement; CSF = corticosteroid free; HEMI = histologic-endoscopic mucosal improvement; HEMR = histologic-endoscopic mucosal remission; mNRI = modified NRI; NRI = nonresponder imputation. Multiple imputation with 100 imputed data sets was used for patients sporadically missing the endpoint data.  * See manuscript section Methods – Outcome Measures for specific endpoint definitions.  ^a^ Sporadic missing is defined as patients who are missing data needed to assess the endpoint due to reasons other than treatment discontinuation.  ^b^ Percentage of response is calculated using Rubin’s rules to combine multiple imputation datasets.  ^c^ Confidence intervals are constructed using Rubin’s rules to combine multiple imputation datasets. Standard errors for each imputed dataset are calculated using the asymptotic method, without continuity correction. | | | | | |

**Supplemental Table 4. LUCENT-3 Extended Induction Rates at 104 Weeks of Continuous Treatment in LUCENT-2 Week 52 Responders, NRI, mNRI, OC**

| **Endpoint** | **W52 Subgroup (N)** | **NRI n (%) [95% CI^a^]** | **mNRI^b,c^ (%)^d^ [95% CI]^e^** | **OC**  **n/N (%) [95% CI^a^]** |
| --- | --- | --- | --- | --- |
| Clinical remission | Responder (81) | 28 (34.6) [25.1, 45.4] | 45.4 (33.9, 56.9) | 28/60 (46.7) [34.6, 59.1] |
| Clinical response | Responder (81) | 55 (67.9) [57.1, 77.1] | 81.9 (72.8, 91.0) | 55/60 (91.7) [81.9, 96.4] |
| Symptomatic remission | Responder (81) | 51 (63.0) [52.1, 72.7] | 76.5 (66.7, 86.3) | 51/60 (85.0) [73.9, 91.9] |
| The modified intent-to-treat population was used with mNRI methods for missing data. Responders: ≥30% and 2-point decrease from baseline in the composite clinical endpoint of the sum of ES, SF, and RB subscores, and RB=0 or 1, or ≥1 pt decrease from baseline. Remitters: MMS SF=0 or SF=1 with ≥1-point decrease from baseline; RB=0; ES=0 or 1. Symptomatic remission: SF=0 or SF=1 with ≥1-point decrease in MMS from baseline; RB=0. Sporadic missing is defined as patients who are missing data needed to assess the endpoint due to reasons other than treatment discontinuation. For mNRI, multiple imputation with 100 imputed data sets was used for patients sporadically missing the endpoint data.  Abbreviations: CI = confidence interval; ES = endoscopic subscore; MMS=modified Mayo score; mNRI = modified NRI; NRI = nonresponder imputation; OC = observed case; RB = rectal bleeding; SF = stool frequency.  ^a^ Response confidence intervals are constructed using Wilson method, without continuity correction.  ^b^ Because multiple imputation uses multiple estimates, there is no end result of number of patients in the analyses for mNRI, but rather the estimated proportion from the model.  ^c^ Clinical remission, clinical response, and symptomatic remission missing data, n (%): Treatment discontinuation = 8 (9.9%); Sporadic missing = 13 (16.0%) each.  ^d^ Percentage of response is calculated using Rubin’s rules to combine multiple imputation datasets.  ^e^ Confidence intervals are constructed using Rubin’s rules to combine multiple imputation datasets. Standard errors for each imputed dataset are calculated using the asymptotic method, without continuity correction. | | | | |

**Supplemental Figure 1. LUCENT-3 Rates at 104 Weeks of**

**Continuous Treatment in LUCENT-2 Week 52 Responders and Remitters by Biologic Failed**

**and Not Failed Treatment Status for A: Alternate Clinical Remission, B: Corticosteroid-free Remission, C: Endoscopic Remission, D: HEMI, E: Bowel Urgency CMI, F: Bowel Urgency Remission, NRI**

The modified intent-to-treat population was used with NRI methods for missing data. Responders: ≥30% and 2-point decrease from baseline in the composite clinical endpoint of the sum of ES, SF, and RB subscores, and RB=0 or 1, or ≥1-point decrease from baseline. Remitters: MMS SF=0 or SF=1 with ≥1-point decrease from baseline; RB=0; ES=0 or 1. Biologic failed = biologic failed patients at LUCENT-1 induction baseline; prior inadequate response, loss of response, or intolerance to biologic therapy or Janus kinase inhibitors (tofacitinib). Not biologic failed = not biologic failed patients at LUCENT-1 induction baseline; patients not meeting biologic failed definition. Alternate clinical remission: SF=0 or SF=1; RB=0; and ES=0 or 1 (excluding friability). Endoscopic remission: ES=0 or 1 (excluding friability). Bowel urgency remission: Urgency NRS=0 or 1. Abbreviations: CI=confidence interval; CMI=clinically meaningful improvement (change from baseline in Urgency NRS ≥3 in patients with Urgency NRS ≥3 at induction baseline); ES=endoscopic subscore; HEMI=histologic-endoscopic mucosal improvement (Geboes ≤3.1 + ES=0 or 1 [excluding friability]); MMS=modified Mayo score; NRI=nonresponder imputation; NRS=numeric rating scale; RB=rectal bleeding; SF=stool frequency.

**Supplemental Figure 2. LUCENT-3 Rates at 104 Weeks of Continuous Treatment in LUCENT-2 Week 52 Responders and Remitters for A: Alternate Clinical Remission, B: Endoscopic Remission, NRI, mNRI, OC**

The modified intent-to-treat population was used with NRI, mNRI, and OC methods used for missing data. Responders: ≥30% and 2-point decrease from baseline in the composite clinical endpoint of the sum of ES, SF, and RB subscores, and RB=0 or 1, or ≥1-point decrease from baseline. Remitters: MMS SF=0 or SF=1 with ≥1-point decrease from baseline; RB=0; ES=0 or 1. Alternate clinical remission: SF=0 or SF=1; RB=0; and ES=0 or 1 (excluding friability). Endoscopic remission: ES=0 or 1 (excluding friability). Abbreviations: CI=confidence interval; ES=endoscopic subscore; MMS= modified Mayo score; mNRI=modified NRI; NRI=nonresponder imputation; OC=observed case; RB=rectal bleeding; SF=stool frequency. For the mNRI population: For W52 responders for endpoints, n (%): Treatment discontinuation = 23 (9.6) and sporadic missing = 32 (13.4) for alternate clinical remission; treatment discontinuation = 23 (9.6) and sporadic missing = 5 (2.1) for endoscopic remission. For W52 remitters, n (%): Treatment discontinuation = 14 (9.1); and sporadic missing = 20 (13.0) for alternate clinical remission; treatment discontinuation = 14 (9.1) and 4 (2.6%) for endoscopic remission.

**Supplemental Figure 3. LUCENT-3 Maintenance Rates at 104 Weeks of Continuous Treatment in**

**LUCENT-2 Week 52 Endpoint Remitters for A: Symptomatic Remission, B: HEMR, C: Bowel Urgency Remission, NRI, mNRI, OC**


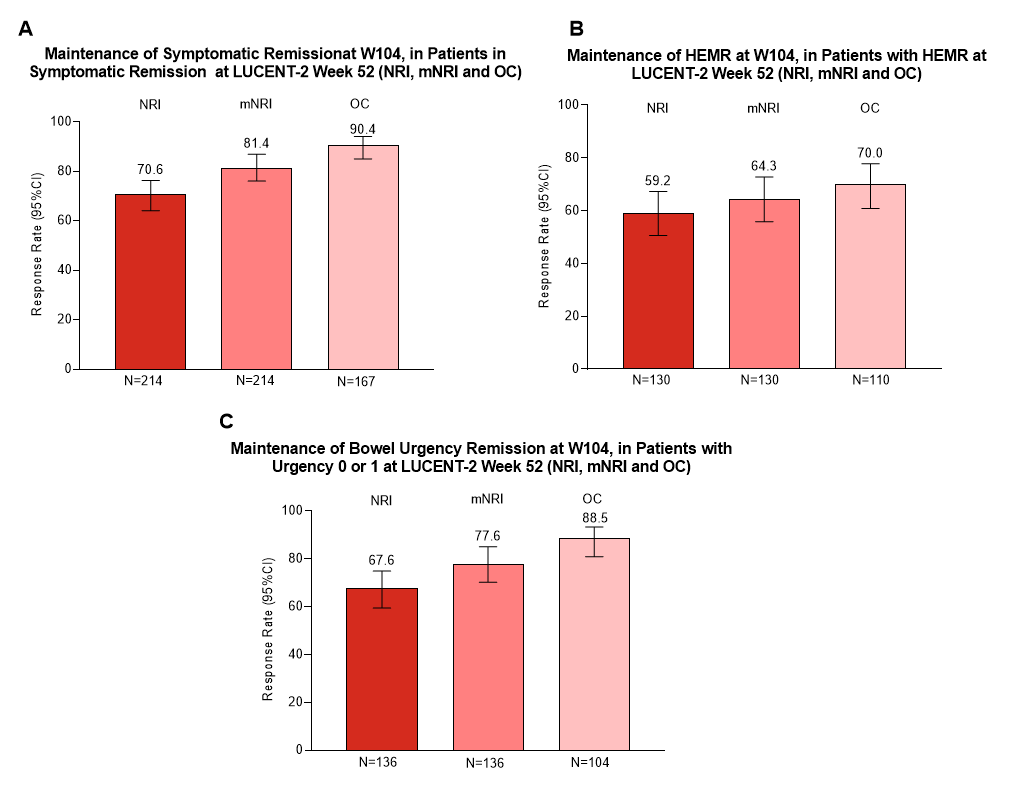


The modified intent-to-treat population was used with NRI, mNRI, and OC methods used for missing data. Symptomatic remission: SF=0 or SF=1 with ≥1-point decrease in MMS from baseline; RB=0. Bowel urgency remission: Urgency NRS=0 or 1. Abbreviations: CI=confidence interval; ES=endoscopic subscore; HEMR=histologic-endoscopic mucosal remission (Geboes ≤2B.0 + ES=0 or 1 [excluding friability]); MMS=modified Mayo score, mNRI=modified NRI; NRI=nonresponder imputation; NRS=numeric rating scale; OC=observed case; RB=rectal bleeding; SF=stool frequency. For the mNRI population: Maintenance of symptomatic remission, n (%): Treatment discontinuation = 20 (9.3); Sporadic missing = 27 (12.6); Maintenance of HEMR, n (%): Treatment discontinuation = 11 (8.5); Sporadic missing = 9 (6.9); Maintenance of bowel urgency remission, n (%): Treatment discontinuation = 15 (11.0); Sporadic missing: 17 (12.5).

**Supplemental Figure 4. LUCENT-3 Maintenance Rates at 104 Weeks of Continuous Treatment in**

**LUCENT-2 Week 52 Endpoint Responders and Remitters by Biologic Failed and Not Failed Treatment Status for A: Clinical Response, B: Clinical Remission, C: Symptomatic Remission, D: HEMR, E: Bowel Urgency Remission, NRI~~, mNRI, OC~~**

The modified intent-to-treat population was used with NRI methods for missing data. For each panel, only the patients who met the noted endpoint at W52 (W40 LUCENT-2) are included in the analyses to ascertain durable maintenance of that endpoint from 52 to 104 weeks of treatment. Responders: ≥30% and 2-point decrease from baseline in the composite clinical endpoint of the sum of ES, SF, and RB subscores, and RB=0 or 1, or ≥1 pt decrease from baseline. Remitters: MMS SF=0 or SF=1 with ≥1-point decrease from baseline; RB=0; ES=0 or 1. Biologic failed = biologic failed patients at LUCENT-1 induction baseline; prior inadequate response, loss of response, or intolerance to biologic therapy or Janus kinase inhibitors (tofacitinib). Not biologic failed = not biologic failed patients at LUCENT-1 induction baseline; patients not meeting biologic failed definition. Symptomatic remission = SF=0 or SF=1 with ≥1-point decrease in MMS from baseline; RB=0. Bowel urgency remission = Urgency NRS=0 or 1 in patients with Urgency NRS ≥3 at induction baseline. Abbreviations: CI=confidence interval; ES=endoscopic subscore; HEMR=histologic-endoscopic mucosal remission (Geboes ≤2B.0 + ES=0 or 1 [excluding friability]); MMS= modified Mayo score; NRI=nonresponder imputation; NRS=numeric rating scale; RB=rectal bleeding; SF=stool frequency; W=week.

**Supplemental Figure 5. LUCENT-3 Extended Induction Rates at 104 Weeks of Continuous Treatment in LUCENT-2 Week 52 Responders by Biologic Failed and Not Failed Treatment Status for A: Clinical Remission, B: Corticosteroid-free Remission, C: Symptomatic Remission, NRI**

The modified intent-to-treat population was used with NRI methods for missing data. Remitters: MMS SF=0 or SF=1 with ≥1-point decrease from baseline; RB=0; ES=0 or 1. Biologic failed = biologic failed patients at LUCENT-1 induction baseline; prior inadequate response, loss of response, or intolerance to biologic therapy or Janus kinase inhibitors (tofacitinib). Not biologic failed = not biologic failed patients at LUCENT-1 induction baseline; patients not meeting biologic failed definition. CSF remission: clinical remission with no corticosteroid use for ≥12 weeks. Symptomatic remission = SF=0 or SF=1 with ≥1-point decrease in MMS from baseline; RB=0. Abbreviations: CI=confidence interval; CSF=corticosteroid free; ES=endoscopic subscore; MMS= modified Mayo score; NRI=nonresponder imputation; RB=rectal bleeding; SF=stool frequency.

**Supplemental Figure 6. LUCENT-3 Extended Induction Rates at 104 Weeks of Continuous Treatment in LUCENT-2 Week 52 Responders by Biologic Failed and Not Failed Treatment Status for A: Endoscopic Remission, B: HEMI, C: HEMR, NRI**

The modified intent-to-treat population was used with NRI methods for missing data. Biologic failed = biologic failed patients at LUCENT-1 induction baseline; prior inadequate response, loss of response, or intolerance to biologic therapy or Janus kinase inhibitors (tofacitinib). Not biologic failed = not biologic failed patients at LUCENT-1 induction baseline; patients not meeting biologic failed definition. Endoscopic remission: ES=0 or 1 (excluding friability). Abbreviations: CI=confidence interval; ES=endoscopic subscore; HEMI=histologic-endoscopic mucosal improvement (Geboes ≤3.1 + ES=0 or 1 [excluding friability]); HEMR=histologic-endoscopic mucosal remission (Geboes ≤2B.0 + ES=0 or 1 [excluding friability]); NRI=nonresponder imputation.

**Supplemental Figure 7. LUCENT-3 Extended Induction Rates at 104 Weeks of Continuous Treatment in LUCENT-2 Week 52 Responders by Biologic Failed and Not Failed Treatment Status for A: Bowel Urgency CMI, B: Bowel Urgency Remission, NRI**

The modified intent-to-treat population was used with NRI methods for missing data. Biologic failed = biologic failed patients at LUCENT-1 induction baseline; prior inadequate response, loss of response, or intolerance to biologic therapy or Janus kinase inhibitors (tofacitinib). Not biologic failed = not biologic failed patients at LUCENT-1 induction baseline; patients not meeting biologic failed definition. Bowel urgency remission = Urgency NRS=0 or 1 in patients with Urgency NRS ≥3 at induction baseline. Abbreviations: NRI=nonresponder imputation; NRS=numeric rating scale.

**Supplemental Figure 8. LUCENT-3 Rates at 104 Weeks of Continuous Treatment in LUCENT-2 Week 52 Responders and Remitters for A: IBDQ Change from Baseline, B: IBDQ Response (IBDQ Total Score ≥16-point Improvement), NRI**
The modified intent-to-treat population was used with modified baseline observation carried forward and NRI methods for missing data for continuous and categorical endpoints, respectively. Baseline was defined as the last non-missing assessment recorded on or prior to the date of the first study drug administration at Week 0 in LUCENT-1 induction study. A) Change from baseline in IBDQ total and domain scores; continuous data using LSM analysis methodology. B) IBDQ response rates (≥16-point improvement from baseline; categorical data). Responders: ≥30% and 2-point decrease from baseline in the composite clinical endpoint of the sum of ES, SF, and RB subscores, and RB=0 or 1, or ≥1-point decrease from baseline. Remitters: MMS SF=0 or SF=1 with ≥1-point decrease from baseline; RB=0; ES=0 or 1. Biologic failed = biologic failed patients at LUCENT-1 induction baseline; prior inadequate response, loss of response, or intolerance to biologic therapy or Janus kinase inhibitors (tofacitinib). Not biologic failed = not biologic failed patients at LUCENT-1 induction baseline; patients not meeting biologic failed definition. Abbreviations: ANCOVA=analysis of covariance; ES=endoscopic subscore; IBDQ=Inflammatory Bowel Disease Questionnaire; LSM=least-squares mean; mBOCF=modified baseline observation carried forward; mITT=modified intent-to-treat; MMS=modified Mayo score; NRI=nonresponder imputation; RB=rectal bleeding; SE=standard error; SF=stool frequency.

**Supplemental Content 1. Missing Data Handling and Importance of Data Interpretation Based Upon Analytical Method**

To limit patient burden, the LUCENT-3 long-term extension study utilized paper diaries filled out once per month within a 24-hour period instead of the ongoing daily electronic digital diaries used in LUCENT-1 and LUCENT-2. At visits that included yearly endoscopies, such as Week 104, patients were asked to fill out the paper diary for the 7 days prior to the endoscopy. While lowering the frequency that patients were required to complete their diary reduced patient burden, it also led to fewer endpoints for analysis, less robust over-time data for stool frequency and rectal bleeding, and less stringent patient follow-through with symptom data entry compared with the LUCENT-1 and LUCENT-2 studies.

There were 2 forms of “missingness”: (1) treatment discontinuation, and (2) sporadic missingness such as due to missed visits or failing to fill out sufficient diary days. As an example, for W104 (LUCENT-3 W52) symptomatic remission, there was a 9.6% and 9.1% patient treatment discontinuation and an additional 13.4% and 13.0% of patients with sporadic missing data among the maintenance responders and remitters, respectively (Supplemental Table 3). Compared with symptom data, sporadic missing endoscopy data were much less of an issue, occurring for only 5 overall patients.

Per the original LUCENT-3 statistical analysis plan, the a priori primary analysis approach was nonresponder imputation (nonresponder imputation). Nonresponder imputation is a conservative analytical approach and treats patients with missing data as nonresponders. This is biased to show low remission/response rates because many of the patients with sporadic missing data could be responders. An observed case approach was also planned; however, observed case only includes patients without missing data, and is biased to show high remission/response rates given it excludes patients with treatment discontinuation who would generally be considered as nonresponse.

A third analysis approach, modified nonresponder imputation, was used as a balance between the two extremes of nonresponder imputation - and observed case-based analyses. The modified nonresponder imputation approach uses multiple imputation for the sporadic missing data but counts treatment discontinuation as nonresponse, showing higher response/remission proportions than nonresponder imputation, but with consistent, expected relationships between endpoints such as clinical response rates higher than endoscopic remission rates, unlike response rates from the nonresponder imputation approach. Together with the pre-planned analyses, these analyses provide important supportive information, but are not appropriate for the small subgroup populations, which is why the efficacy-by-biofailed status was not analyzed for modified nonresponder imputation. Additionally, the modified nonresponder imputation method includes modeling that involved biofailed status, an additional reason modified nonresponder imputation was not used for the subgroup analyses.

The addition of the modified nonresponder imputation analyses is important because approximately 25% of patients are missing at W104 for the interim LUCENT-3 analyses, with approximately 10% missing due to discontinuations and the other 15% sporadically missing (missing at random). The sporadic missing data are generally patient-reported symptom data, and tagging these patients as nonresponders, when that is unlikely, may have led to lower response rates for endpoints focused on symptomatic data. In fact, missing values in LUCENT-3 for the nonresponder imputation analyses did affect the rates of symptomatic remission, which was especially noticeable in the maintenance remitters analyses, where an inversion of the usual rates of symptomatic versus endoscopic outcomes was observed. Clinical response rates and symptomatic remission rates were lower than expected when compared to endpoints such as endoscopic remission. This finding is irregular since a clinical response rate or symptomatic remission rate with a lower threshold bar to meet should have higher attainment rates than an endpoint such as endoscopic remission. The impact was likely larger in the remission analyses because of the lower total patient number, allowing missing data to have a larger impact. Missing symptomatic data affected clinical response and remission rates that include stool frequency and rectal bleeding as part of the composite endpoint but did not affect endoscopic and histologic endpoints in the same way. The analyses with the modified nonresponder imputation approach addressed this issue by using multiple imputation for the sporadically missing data, leading to clinical response and symptomatic remission rates that were higher than endoscopic remission rates, as would be clinically expected and as observed across all clinical trial research for UC studies, including LUCENT-1 and LUCENT-2.
